# Effects of Systemic Anticancer Treatment on Cardiorespiratory Fitness: A Systematic Review and Meta-Analysis

**DOI:** 10.1016/j.jaccao.2024.11.004

**Published:** 2025-01-14

**Authors:** Sara H. Johansen, Torbjørn Wisløff, Elisabeth Edvardsen, Sofie T. Kollerud, Johanne S.S. Jensen, Ginika Agwu, Konstantina Matsoukas, Jessica M. Scott, Tormod S. Nilsen

**Affiliations:** a Department of Physical Performance, The Norwegian School of Sport Sciences, Oslo, Norway; b Health Services Research Unit, Akershus University Hospital, Lørenskog, Norway; c Department of Pulmonary Medicine, Oslo University Hospital, Oslo, Norway; d Memorial Sloan Kettering Cancer Center, New York, New York, USA

**Keywords:** alkylating therapy, anthracycline, cancer, cardiorespiratory fitness, peak oxygen consumption, physiological determinants, survivorship, systemic anticancer treatment, treatment, V_O2_ and exercise

## Abstract

**Background:**

Poor cardiorespiratory fitness (CRF) is associated with a higher symptom burden and an increased prevalence of long-term treatment–related cardiovascular disease risk factors in cancer survivors. However, the magnitude of systemic therapy–related CRF impairment remains unclear.

**Objectives:**

The aim of this study was to evaluate the effects of systemic anticancer treatment on CRF and identify physiological determinants underpinning CRF impairment.

**Methods:**

A systematic literature search was performed in PubMed, Embase, CINAHL, SPORTDiscus, and the Cochrane Library. The primary endpoint was the change in CRF, measured by peak oxygen consumption (Vo_2peak_), from before to after systemic treatment. Secondary endpoints included post-treatment differences in Vo_2peak_ between cancer survivors and noncancer control subjects, along with physiological determinants of Vo_2peak_. Two meta-regressions were conducted to examine the association between CRF and cardiac output and arteriovenous oxygen difference.

**Results:**

A total of 44 studies were included, comprising 27 prospective trials (61%; n = 1,234 cancer survivors, median age 52.4 years) and 17 cross-sectional studies (39%; n = 1,372 cancer survivors, median age 54.0 years; n = 1,923 noncancer control subjects, median age 56.0 years). Systemic anticancer treatment was associated with a significant decrease in Vo_2peak_ (weighted mean difference −2.13 mL·kg^−1^·min^−1^; 95% CI: −2.76 to −1.50 mL·kg^−1^·min^−1^). No significant differences were observed between patient subgroups (esophagogastric, breast, and colon or rectal cancers). At a median follow-up of 2 years (range: 6 weeks to 12 years) post-therapy, cancer survivors had a significantly lower Vo_2peak_ (weighted mean difference −6.39 mL·kg^−1^·min^−1^; 95% CI: −7.60 to −5.18 mL·kg^−1^·min^−1^) compared with noncancer control subjects. Reduced arteriovenous oxygen difference was associated with lower Vo_2peak_ (β = 2.55; 95% CI: 2.05-3.06; *P* < 0.001).

**Conclusions:**

Systemic anticancer treatment leads to substantial and sustained impairments in CRF.

Cardiorespiratory fitness (CRF), measured by peak oxygen consumption (Vo_2peak_), provides an objective indicator of overall cardiovascular capacity.^1^ In patients with cancer, low Vo_2peak_ is associated with a higher symptom burden^2^ and an increased prevalence of long-term treatment–related cardiovascular disease risk factors^3^ and is a strong, independent predictor of cancer, cardiovascular, and all-cause mortality.^4^^,^^5^ Findings from several studies suggest that systemic anticancer treatment can lead to significant acute and chronic reductions in Vo_2peak_.^5^, ^6^, ^7^ However, most existing studies have focused on breast cancer, lacked longitudinal assessments, or relied on estimated rather than directly measured Vo_2peak_. Thus, the broader effects of systemic anticancer therapies on Vo_2peak_ remain inadequately understood.

Physiological determinants of Vo_2peak_ include both central factors, such as reduced convective O_2_ transport, and peripheral factors, such as decreased diffusive O_2_ transport and oxidative capacity in skeletal muscle.^8^ Although O_2_ transport (eg, cardiac output) is often a limiting factor for Vo_2peak_ in noncancer settings,^9^ anticancer treatments can cause both short- and long-term adverse effects that may create limitations at various points along the cardiopulmonary-muscle axis.^10^ There is a pressing need to characterize the pathophysiology of poor Vo_2peak_ in patients with cancer to guide the development of targeted interventions.

The aim of this systematic review and meta-analysis was to evaluate the effects of systemic anticancer treatment on Vo_2peak_ in adults with cancer. Secondary objectives included comparing differences in Vo_2peak_ between cancer survivors and noncancer control subjects in cross-sectional studies and evaluating physiological determinants of Vo_2peak_.

## Methods

### Search strategy and selection criteria

A comprehensive literature search was conducted by a research informationist (K.M.) from database inception to January 20, 2023. The systematic literature review included searches in PubMed (National Library of Medicine), Embase, CINAHL, SPORTDiscus, and the Cochrane Library. The search strategy used terms related to systemic anticancer treatment, CRF, and CRF determinants (Supplemental Appendix 1). An updated search was conducted on January 17, 2024, to capture newly published trials.

Randomized (limited to nonintervention control groups) or nonrandomized trials, prospective cohort studies with pre- and post-treatment assessments, and cross-sectional studies with post-treatment assessments that included a noncancer control group for reference values were considered eligible if they met the following criteria: 1) included adult patients (>18 years of age) diagnosed with adult-onset cancer, regardless of stage, or adult survivors of any (childhood) cancer who had received systemic anticancer treatment (eg, chemotherapy, stem cell transplantation, endocrine agents, targeted or biological agents, and/or immune checkpoint inhibitors); and 2) directly measured Vo_2peak_ (eg, using ergospirometry) in milliliters per kilogram per minute before and/or after systemic therapy. Studies reporting 1 or more physiological determinants of Vo_2peak_ were included in a subgroup analysis. Cancer survivors were defined as individuals from the time of cancer diagnosis until the end of life.^11^ For cases that reported Vo_2peak_ solely in absolute values (L/min), a request for additional data was sent to the corresponding author.

Exclusion criteria included studies that did not report group central tendencies and distributions; used submaximal and/or indirect Vo_2peak_ tests; lacked a noncancer reference group for cross-sectional studies; or were abstracts, systematic reviews, protocols, duplicates, or not written in English. Studies were also excluded if there was no response to requests for additional data. To ensure transparency and adherence with reporting standards, this systematic review and meta-analysis was preregistered in the International Prospective Register of Systematic Reviews (CRD42023361788) and prepared in accordance with the Preferred Reporting Items for Systematic Reviews and Meta-Analyses guidelines (Supplemental Appendix 2 and Supplemental Appendix 3).^12^

### Study selection and assessment of risk for bias

Screening of potential studies involved evaluating titles, abstracts, and full texts according to predefined inclusion criteria by 2 independent assessors (S.H.J. and S.T.K.) using Covidence systematic review software (Veritas Health Innovation). In cases of disagreement, a third assessor (T.S.N.) was consulted to reach consensus. Data extraction and assessment of risk for bias were carried out using a standardized data extraction template by a team of 4 independent reviewers (S.H.J., T.S.N., J.S.S.J., and G.A.), with the role of the second reviewer distributed among T.S.N., J.S.S.J., and G.A. Data were extracted from the primary reports and supplemental materials (Supplemental Tables 1 to 4, Supplemental Appendix 4 and 5).

### Data synthesis and statistical analysis

All analyses were performed in R version 4.3.2 (R Foundation for Statistical Computing) using RStudio version 2023.06.0 (Posit). A random-effects meta-analysis with inverse variance weighting was used to calculate the weighted mean difference (WMD) for Vo_2peak_, using the function metacont from the meta package. Standardized mean differences were calculated using Hedges’s *g*. Heterogeneity was calculated using both the *I*^2^ and τ^2^ statistics, as defined by Schwarzer et al^13^ (Supplemental Funnel plots, Supplemental Appendix 6, Supplemental Figures 1 and 2). Publication bias was evaluated using funnel plots. Because of variability in reporting, participant age and time since treatment cessation were summarized as the study-level median (range) across studies.

Three a priori subgroup analyses were conducted to investigate the impact of treatment type, primary cancer site, and whether the effects differed on the basis of follow-up time. However, because of insufficient reporting, variability in treatment regimens, and small sample sizes, the analysis for treatment type was not feasible. Subgroup analyses were performed using mixed-effects meta-regression, with Vo_2peak_ as the outcome, incorporating cancer type or follow-up time as predictors, using the function metareg from the metafor package.

For studies in which all relevant information was available (Vo_2peak_, cardiac output, and arteriovenous oxygen [a-vO_2_] difference), separate analyses were performed with cardiac output and a-vO_2_ difference as additional predictors, but these were not included simultaneously because of the limited number of studies. When a study’s SD was not reported, it was calculated from the CI or SE reported. The 95% CIs depicted for each study in the forest plot of this meta-analyses are based on the normal distribution and may differ slightly from those reported in the original studies. However, this variation does not affect the pooled results.

Long-term survivors were defined as individuals who had completed systemic therapy more than 5 years prior. Forest plots were used to display the results of the meta-analysis, including the mean difference for each study and the WMD with 95% CIs. For each subgroup analysis, a meta-regression was conducted, as described earlier, to evaluate potential differences between groups. These meta-analyses were performed similarly to standard regression, with the post-treatment value as the outcome, controlling for baseline and incorporating the factor of interest (diagnosis or long-term survivor status).

## Results

**Figure 1:**
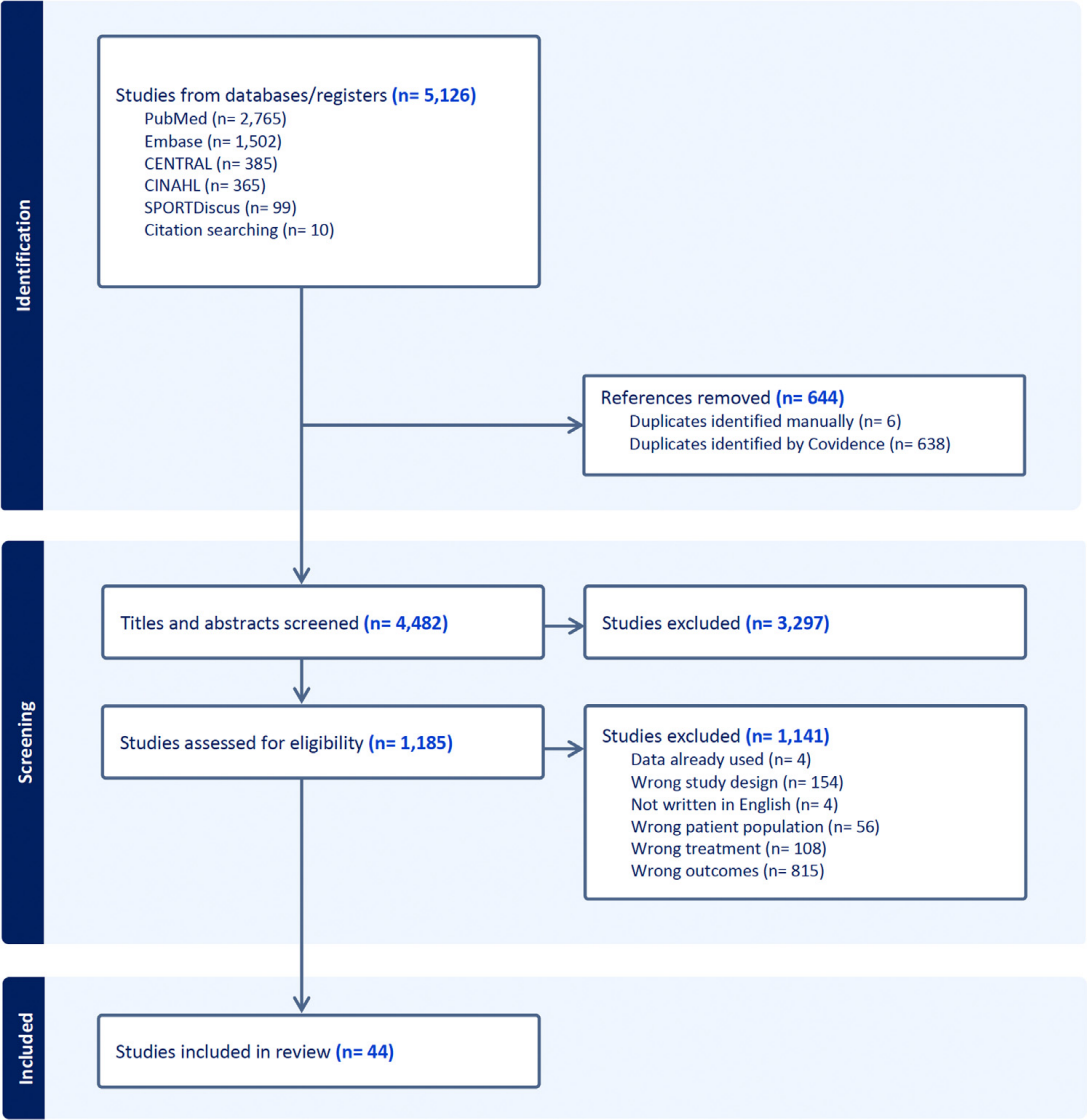
Flowchart According to Preferred Reporting Items for Systematic Reviews and Meta-Analyses Guidelines Flowchart illustrating the study selection process following the Preferred Reporting Items for Systematic Reviews and Meta-Analyses guidelines, including the number of records identified, screened, and included.

A total of 5,126 records were identified, with 644 duplicates removed. This left 4,482 unique records for screening, 44 of which met the inclusion criteria and were included in the analyses (Figure 1).

### Study and population characteristics

The 44 included studies spanned publication years from 1994 to 2023. Among these, 27 studies (61%) were prospective trials (Supplemental Appendix 7, Supplemental Table 5)^14^, ^15^, ^16^, ^17^, ^18^, ^19^, ^20^, ^21^, ^22^, ^23^, ^24^, ^25^, ^26^, ^27^, ^28^, ^29^, ^30^, ^31^, ^32^, ^33^, ^34^, ^35^, ^36^, ^37^, ^38^, ^39^, ^40^ with pre- and post-treatment assessments, and 17 studies (39%) were cross-sectional (Supplemental Appendix 7, Supplemental Table 6), comparing post-treatment Vo_2peak_ in cancer survivors with noncancer control subjects^3^^,^^41^, ^42^, ^43^, ^44^, ^45^, ^46^, ^47^, ^48^, ^49^, ^50^, ^51^, ^52^, ^53^, ^54^, ^55^, ^56^ (Table 1). Of the included studies, 21 (48%) focused on breast cancer, and 40 (91%) primarily used chemotherapy as the treatment modality. Across all studies, there were 2,606 cancer survivors (median age 52.7 years; range: 19-72.5 years) and 1,923 noncancer control subjects (median age 56.0 years; range: 22-67 years). In the prospective trials (n = 1,234), the median treatment duration was 13 weeks (range: 7-27 weeks), though 29% of trials did not report treatment duration. Cross-sectional studies included 1,372 cancer survivors (median time post-treatment 2 years; range: 6 weeks-12 years) and 1,923 noncancer control subjects. Four trials^3^^,^^44^^,^^49^^,^^54^ (n = 114 cancer survivors, n = 50 noncancer control subjects) were included in the analysis of physiological determinants of Vo_2peak_.

**Table 1:** Characteristics of the Included Trials (N = 44)

Study design	
Cross-sectional study	17 (39)
Randomized controlled trial	14 (32)
Prospective cohort study	10 (23)
Nonrandomized controlled trial	3 (7)
Region of origin	
Canada	11 (25)
United States	10 (23)
Australia	6 (14)
United Kingdom	6 (14)
Denmark	2 (5)
France	2 (5)
Brazil	1 (2)
Egypt	1 (2)
Germany	1 (2)
Sweden	1 (2)
Switzerland	1 (2)
Taiwan	1 (2)
the Netherlands	1 (2)
Year of publication	
1994-2010	4 (9)
2010-2019	19 (43)
2020-2023	21 (48)
Sample size	
<20	16 (36)
21-50	18 (41)
>50	10 (23)
Total number of cancer survivors	2,606
Total number of noncancer control subjects	1,923
Sample size by diagnosis	
Mixed diagnosis	1,154 (44)
Breast cancer	622 (24)
Esophagogastric	230 (9)
Leukemia/lymphoma	372 (14)
Lung	126 (5)
Colon/rectal	60 (2)
Prostate	26 (1)
Head and neck/CNS	16 (1)
Age of cancer survivors, y	52.7 (19-72.5)
Age of noncancer control subjects, y	56.0 (22-67)
Cancer site^a^	
Breast	21 (48)
Mixed diagnosis	6 (14)
Esophageal/gastric	5 (11)
Colon/rectal	3 (7)
Lung	3 (7)
Leukemia/lymphoma	4 (9)
Head and neck/CNS	1 (2)
Prostate	1 (2)
Systemic treatment^b^	
Chemotherapy	40 (91)
Targeted/biological agents	7 (16)
Endocrine therapies^c^	3 (7)
Hematopoietic stem cell transplantation	2 (5)
Regimens that could not be categorized	1 (2)

Values are n (%) or median (range).

CNS = central nervous system.

a Studies may be counted in multiple categories because of subgroup analysis.

b Studies may be counted in multiple categories because of multimodal regimens.

c Studies with endocrine agents as the only systemic treatment received.

### Assessments of risk for bias

Attrition and reporting bias were low in 12 (71%) and 14 (82%) of the 17 clinical trials, respectively (Supplemental Table 2). Among the cross-sectional studies, 3 (18%) achieved participation rates exceeding 50%, whereas 11 (65%) did not report participation rates (Supplemental Table 4, Q3). In the prospective cohort trials, loss to follow-up was <20% in 3 studies (30%) (Supplemental Table 3, Q9). The funnel plots showed relative symmetry, indicating low risk for publication bias in the included studies (Supplemental Figures 1 and 2).

### Effect of systemic anticancer treatment on Vo_2peak_

**Figure 2:**
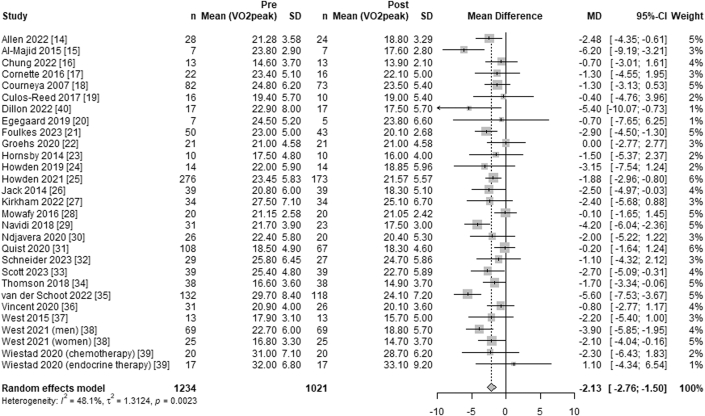
Forest Plot of the Effect of Systemic Anticancer Treatment on Vo_2peak_ Forest plot depicting the effects of systemic anticancer treatments on peak oxygen consumption (Vo_2peak_) in a meta-analysis of studies with pre- and post-treatment assessments. The summary estimate was calculated using a random-effects model, with the mean differences and corresponding 95% CIs. The overall summary effect is represented by a diamond.

**Figure 3:**
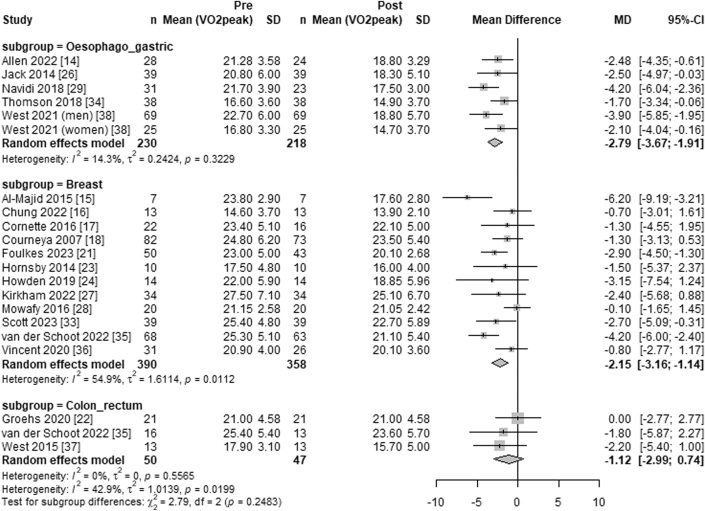
Forest Plot of the Effect of Systemic Anticancer Treatment on Vo_2peak_ by Cancer Diagnosis Forest plot illustrating the effects of systemic anticancer treatments on peak oxygen consumption (Vo_2peak_), stratified by cancer diagnosis subgroups. Summary estimates for each subgroup were calculated using a random-effects model, with mean differences and 95% CIs shown as bars. The overall effect within each subgroup is represented by the corresponding diamond.

Overall, systemic anticancer treatment was associated with a significant decrease in Vo_2peak_ (WMD −2.13 mL·kg^−1^·min^−1^; 95% CI: −2.76 to −1.50 mL·kg^−1^·min^−1^; *I*^2^ = 48%) from pre- to post-treatment (Figure 2). Subgroup analysis using meta-regression, with the colon or rectal cancer subgroup as the reference, showed no significant difference in the decline of Vo_2peak_ between treatment for colon or rectal cancer (WMD −1.12 mL·kg^−1^·min^−1^; 95% CI: −2.99 to 0.74 mL·kg^−1^·min^−1^; *I*^2^ = 0%) and esophagogastric cancer (WMD −2.79 mL·kg^−1^·min^−1^; 95% CI: −3.67 to −1.91 mL·kg^−1^·min^−1^; *I*^2^ = 14%) (*P* = 0.46) or breast cancer (WMD −2.15 mL·kg^−1^·min^−1^; 95% CI: −3.16 to −1.14 mL·kg^−1^·min^−1^; *I*^2^ = 55%) (*P* = 0.059) (Figure 3).

### Post-treatment Vo_2peak_ in cancer survivors compared with noncancer control subjects

**Figure 4:**
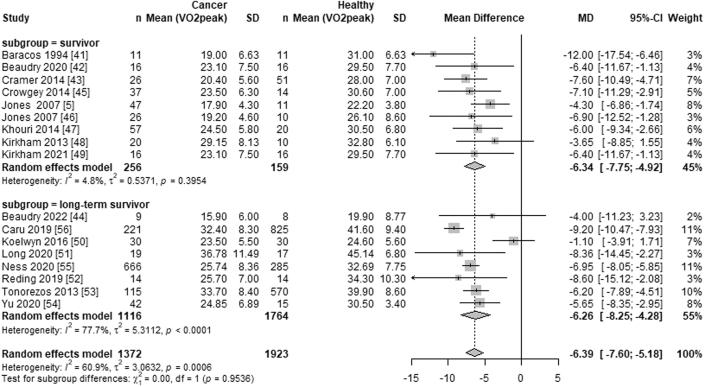
Forest Plot Comparing the Effects of Systemic Anticancer Treatments on Vo_2peak_ Between Cancer Survivors and Noncancer Control Subjects Forest plot comparing the effects of systemic anticancer treatments on peak oxygen consumption (Vo_2peak_) in cancer survivors vs noncancer control subjects. Summary estimates for each follow-up time subgroup were calculated using a random-effects model, with mean differences and 95% CIs shown as bars. Subgroup-level summary effects are represented by diamonds, with the overall effect displayed at the bottom.

After a median of 2 years (range: 6 weeks to 12 years) post-therapy, Vo_2peak_ was significantly lower in cancer survivors (n = 1,372; WMD −6.39 mL·kg^−1^·min^−1^; 95% CI: −7.60 to −5.18 mL·kg^−1^·min^−1^; *I*^2^ = 61%) compared with noncancer control subjects (n = 1,923 participants). The subgroup analysis revealed no differences in Vo_2peak_ impairment between cancer survivors and noncancer control subjects in the short term (median time 14.6 months; range: 6 weeks-34 months post-treatment; WMD −6.34 mL·kg^−1^·min^−1^; 95% CI: −7.75 to −4.92 mL·kg^−1^·min^−1^; *I*^2^ = 5%) compared with the long term (median time 8.4 years; range: 7 to 12 years post-treatment; WMD −6.26 mL·kg^−1^·min^−1^; 95% CI: −8.25 to −4.28 mL·kg^−1^·min^−1^; *I*^2^ = 78%) (*P* = 0.068) (Figure 4).

### Mechanisms underpinning impaired Vo_2peak_

The av-O_2_ difference was indirectly calculated as the ratio of Vo_2peak_ to peak cardiac output across all trials, with cardiac output assessed using stress echography,^44^^,^^54^ impedance cardiography,^3^ and magnetic resonance imaging.^49^ A lower av-O_2_ difference was significantly associated with a lower Vo_2peak_ (β = 2.55; 95% CI: 2.05-3.06; *P* < 0.001). In contrast, no significant association was observed between cardiac output and Vo_2peak_ (β = −0.88; 95% CI: −1.95 to 0.18; *P* = 0.10).

## Discussion

The findings from this meta-analysis indicate that 13 weeks of systemic anticancer treatment results in a weighted mean decline in Vo_2peak_ of approximately 2.1 mL·kg^−1^·min^−1^. Furthermore, Vo_2peak_ remains lower in cancer survivors compared with noncancer control subjects, even years after the completion of treatment (Central Illustration). Given the established association between impaired Vo_2peak_ and adverse clinical outcomes in cancer patients,^57^, ^58^, ^59^ these findings support the recommendation for exercise therapy aimed at preserving and improving Vo_2peak_ during and following cancer treatment.

**Central Illustration:**
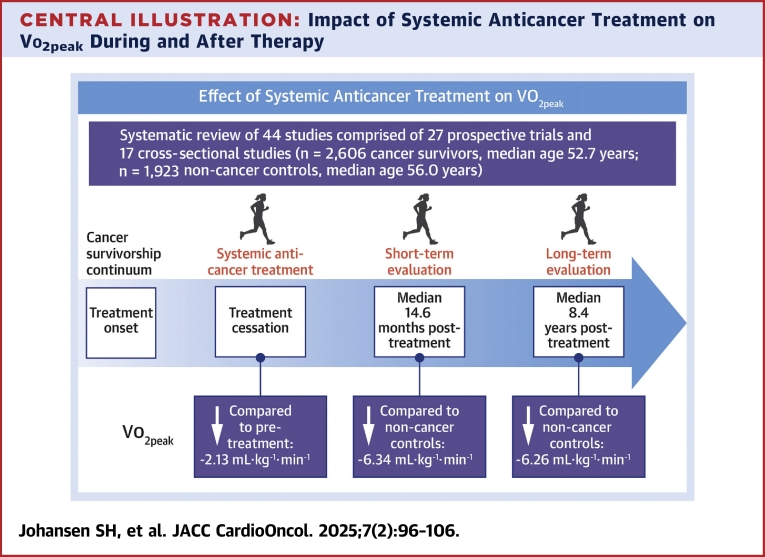
Impact of Systemic Anticancer Treatment on Vo_2peak_ During and After Therapy The illustration depicts the effects of systemic anticancer treatment on cardiorespiratory fitness (CRF) during active treatment and the survivorship phase, compared with noncancer control subjects. It highlights both the acute decline in CRF during treatment and the sustained, persistent decrease among cancer survivors following therapy. Vo_2peak_ = peak oxygen consumption.

To our knowledge, this is the first study to extensively characterize the magnitude of systemic therapy–related impairments across various cancer settings. Most previous studies evaluating CRF responses have focused on a single cancer type. For example, Jones et al^5^ compared Vo_2peak_ in 248 women from 4 cross-sectional breast cancer cohorts with population-based normative data and found Vo_2peak_ levels to be 22% to 33% lower than age- and sex-predicted sedentary values. Similarly, Peel et al^7^ reported that Vo_2peak_ was 25% lower in patients with breast cancer after adjuvant therapy compared with healthy, sedentary women. Our results, drawn from a large, heterogeneous cohort of patients with cancer, significantly extend evidence by providing a comprehensive assessment of systemic therapy–related impairments across multiple cancer types.

Another noteworthy finding was the similar magnitude of Vo_2peak_ decline across subgroups. Specifically, treatment regimens for esophagogastric cancers (commonly treated with cisplatin and oxaliplatin), breast cancers (commonly treated with epirubicin and doxorubicin), and colon and rectal cancers (commonly treated with combination regimens as leucovorin calcium [folinic acid], fluorouracil, and oxaliplatin) resulted in comparable declines in Vo_2peak_. However, a subgroup analysis based on treatment type was not feasible, because of insufficient reporting, as 44% of the total sample comprised participants with mixed cancer diagnoses. Given that heterogeneity in cancer diagnoses and treatment modalities may obscure treatment-specific impacts, further research focused on specific cancer types and comprehensive longitudinal assessments is needed to better understand therapy-specific changes in Vo_2peak_.

In addition to highlighting the decline in CRF during cancer treatment, our findings also revealed lower CRF in long-time cancer survivors compared with noncancer control subjects. Given that Vo_2peak_ typically declines by approximately 10% per decade,^60^ the magnitude of CRF impairment in a typical 50-year-old individual is comparable with that of a 70-year-old individual without a history of systemic cancer treatment. Other studies suggest that cancer survivors treated with systemic therapies are also at risk for accelerated aging processes. For instance, Guida et al^61^ reported that adult childhood cancer survivors, compared with age-matched control subjects, exhibited an aging rate that was 5% faster per year, were biologically 0.6 to 6.44 years older, and aged 5 to 16 years beyond the expected biological age. Collectively, these results support the notion that systemic cancer therapy contributes to accelerated and sustained aging phenotypes.^62^

The direct and indirect adverse effects of anticancer therapy can affect all stages of the O_2_ cascade. However, unlike previous research in noncancer settings, which suggests that blunted cardiovascular O_2_ delivery is primarily responsible for poor Vo_2peak_,^1^ our meta-regression did not identify a significant association between cardiac output and CRF.

Interestingly, we found that lower a-vO_2_ difference values were correlated with lower CRF levels. These finding suggests that systemic anticancer treatment may disproportionately affect the peripheral components of the cardiopulmonary-muscle axis, rather than the central components. However, it is worth noting that variations in methods used to measure cardiac output, such as stress echocardiography, impedance cardiography, and stress magnetic resonance imaging, may have influenced the observed associations. Given that systemic anticancer therapies can affect the entire cardiopulmonary-muscle axis, including pulmonary and vascular function,^63^ with well-established associations between Vo_2peak_ and cardiac output in cancer survivors,^44^^,^^49^ additional research is essential to comprehensively understand the physiological determinants of CRF impairments in this population.

Skeletal muscle deconditioning during systemic anticancer treatment may also contribute to impaired CRF. Mijwel et al^64^ reported a reduction in muscle fiber cross-sectional area, a shift toward a greater proportion of fast-twitch fibers, and decreased mitochondrial content and function. Recent research further suggests a link between chemotherapy and reduced muscle quality, specifically through intermuscular adipose tissue (ie, myosteatosis). For example, Beaudry et al^42^ reported a strong correlation between increased intermuscular adipose tissue content and reduced CRF during chemotherapy for breast cancer. Importantly, longitudinal data have indicated that elevated myosteatosis levels persisted 1 year after chemotherapy in breast cancer survivors.^27^ These findings suggest that systemic cancer treatment may also affect peripheral factors that influence the a-vO_2_ difference.

Strategies to prevent or reverse treatment-associated impairments in Vo_2peak_ are essential. Exercise training is widely considered the most effective intervention to improve Vo_2peak_. Findings from a meta-analysis involving cancer survivors during and after treatment demonstrated that exercise training significantly improved Vo_2peak_ relative to sedentary control subjects.^65^ However, it remains unclear whether initiating exercise during or after systemic therapy is the optimal timing for improving Vo_2peak_. Recent trials involving patients undergoing chemotherapy for breast cancer have indicated that continuous exercise training, both during and after therapy, can lead to clinically meaningful improvements in Vo_2peak_ compared with usual care.^33^^,^^35^

Intriguingly, the magnitude of exercise-induced improvement in Vo_2peak_ may be insufficient to fully mitigate or reverse short-term therapy–related or long-term therapy–related impairments. This suggests that existing national guidelines,^57^, ^58^, ^59^ which recommend generic exercise doses (eg, 3 sessions per week, 30-60 minutes per session at moderate intensity for 12-15 weeks), may be inadequate for enhancing Vo_2peak_ in patients previously treated with systemic therapy. Consequently, there is a critical need to investigate alternative exercise doses, physiologically targeted regimens, and exercise adjuncts such as nutritional or pharmacologic interventions to maximize Vo_2peak_ response among cancer survivors.

### Study limitations

First, our analysis included a heterogeneous sample comprising various cancer diagnoses and treatment regimens. The presence of mixed diagnoses, variability in treatment combinations across studies, and inconsistent reporting complicated the investigation of specific systemic treatment effects on Vo_2peak_. Additionally, the study sample primarily included women with early-stage breast cancer, which necessitates caution when generalizing our findings to other cancer populations. This meta-analysis also incorporated data from various study designs. Although this diversity may influence the interpretation of our findings, it was essential to provide a comprehensive assessment of the available evidence, given the diverse nature of research in this field.

Second, studies included in this analysis generally had relatively small sample sizes. Third, as with all meta-analyses, we are reliant on the data reported in published studies, which may introduce limitations in data quality and completeness.

Finally, the limited number of trials measuring peak cardiac output and a-vO_2_ difference may have influenced the conclusions drawn from the meta-regression. This highlights the need for methodologically rigorous and extensive trials to better understand the physiological mechanisms underlying CRF impairments during and following cancer treatment.

## Conclusions

Systemic anticancer therapy leads to significant and persistent impairments in CRF. Our findings support recommendations for exercise therapy aimed at mitigating and reversing CRF decline during and after cancer therapy.

Perspectives **COMPETENCY IN MEDICAL KNOWLEDGE:** Poor CRF is associated with a higher symptom burden and an increased prevalence of long-term treatment–related cardiovascular disease risk factors in cancer survivors. Our findings confirm that systemic cancer therapy impairs CRF and indicate that cancer survivors have lower CRF levels than noncancer control subjects, underscoring the need for targeted interventions to reduce the long-term risk for severe disease in this population.

**TRANSLATIONAL OUTLOOK:** Further research is essential to achieve a comprehensive understanding of the physiological determinants contributing to persistent CRF impairments in cancer survivors.

## Funding Support and Author Disclosures

Drs Johansen and Nilsen were supported by the Norwegian Cancer Society and AKTIV mot kreft (AKTIV Against Cancer). Dr Scott is supported by the Memorial Sloan Kettering Cancer Center Support Grant/Core Grant (P30 CA008748). All other authors have reported that they have no relationships relevant to the contents of this paper to disclose.
