# Supplementary material for: Effects of Systemic Anticancer Treatment on Cardiorespiratory Fitness: A Systematic Review and Meta-Analysis
Source: JACC CardioOncol. 2025 Jan 14;7(2):96–106. doi: 10.1016/j.jaccao.2024.11.004 (PMC11866419; doi:10.1016/j.jaccao.2024.11.004)
Supplement: Supplemental Material [file mmc1.docx]

## Effects of Systemic Anticancer Treatment on Cardiorespiratory Fitness: A Systematic Review and Meta-Analysis

**Supplemental Materials**

**Supplemental Appendix 1.** Description of Search Methodology.

**Supplemental Appendix 2.** PRISMA 2020 for Abstract Checklist.

**Supplemental Appendix 3.** PRISMA 2020 Checklist.

**Supplemental Appendix 4.** Endpoints and extracted variables.

**Supplemental Appendix 5.** Quality assessment.

**Supplemental Appendix 6.** Funnel plots.

**Supplemental Appendix 7.** Descriptive study characteristics.

**Supplemental Appendix 1. Description of Search Methodology**

Five bibliographic databases - PubMed/MEDLINE (NLM), EMBASE (Elsevier), CINAHL (EBSCO), SPORTDiscus (EBSCO), and Cochrane Library (Wiley)- were originally searched in January 2023 (1/20/2023) and updated in January 2024 (1/17/2024) to identify English language literature (no date restrictions were applied).

The search strategy consisted of keyword terms and controlled vocabulary describing three main concepts: cancer/systemic anticancer treatment, cardiorespiratory fitness (CRF), and determinants of CRF. A methodological search filter was adapted/applied to the search results of PubMed/MEDLINE, EMBASE, and CINAHL (See the full database search below). The literature search initially covered both pre-clinical and clinical trials. However, pre-clinical trials were ultimately excluded during the screening process due to their lack of relevance. Database citation records were imported into the Covidence systematic review software (Veritas Health Innovation, Melbourne, Australia) where duplicate records were removed using automation, with some duplicates identified manually during subsequent screening.

1. Search strategy translated for **PubMed/MEDLINE (NLM)**

|  | Search strategy component concepts |
| --- | --- |
| 1 | Cancer Treatment/Systemic Anticancer Therapy |
|  | (cancer OR oncolog* OR neoplasm* OR carcinom* OR tumor* OR tumour* OR malignan* OR "hematooncological" OR "hemato-oncological" OR "hematologic neoplasms" OR leukemia*) AND ("Antineoplastic Agents"[Mesh] OR "Antineoplastic Combined Chemotherapy Protocols"[Mesh] OR "Antineoplastic Protocols"[Mesh] OR ("Antineoplastic therapy" OR "cancer therapy"[Title/Abstract:~3] OR (cancer* AND "chemotherapy") OR "Antineoplastic treatment" OR "cancer treatment")) |
| 2 | Cardiorespiratory fitness (CRF) |
|  | ("Cardiorespiratory Fitness"[Mesh] OR "Cardiorespiratory Fitness" OR "cardiovascular reserve capacity" OR "cardiorespiratory capacity" OR "cardiovascular capacity" OR "CVRC" OR "aerobic capacity" OR "aerobic fitness" OR "VO2" OR "VO(2)" OR "peak VO2" OR "peak VO(2)" OR "Vo2 peak" OR "VO(2)peak" OR "VO2peak" OR "VO(2peak)" OR "VO 2peak" OR "peak oxygen consumption" OR "peak aerobic capacity" OR "oxygen uptake" OR "oxygen delivery" OR "cardiopulmonary function" OR "ml/kg/min" OR "Oxygen Consumption"[Mesh] OR "oxygen consumption" OR "max VO2" OR "max VO(2)" OR "VO2 max" OR "VO(2)max" OR "VO2max" OR "VO(2max)" OR "VO 2max" OR "maximal aerobic capacity" OR "maximum oxygen consumption" OR "maximum oxygen uptake" OR "cardiopulmonary exercise stress test" OR "cardiopulmonary exercise testing" OR "cardiopulmonary exercise test" OR "CPET" OR "Exercise Test"[Mesh] OR "exercise test" OR "exercise testing" OR "fitness test" OR "Heart Function Tests"[Mesh] OR "Respiratory Function Tests"[Mesh] OR "Anaerobic Threshold"[Mesh] OR "ventilatory anaerobic threshold" OR "Physical Fitness"[Mesh] OR "physical fitness" OR "physical capacity" OR "Physical Endurance"[Mesh] OR "Exercise Tolerance"[Mesh] OR "cardiorespiratory fitness" OR "cardiovascular endurance" OR "cardiovascular response" OR "cardiovascular form" OR "exercise capacity" OR "heart rate recovery" OR "HRR") |
| 3 | Physiological determinants of CRF  (Cardiac function/ Pulmonary function/Gas exchange/ Peripheral factors) |
|  | (("Heart Function Tests"[Mesh] OR "Cardiac Output"[Mesh] OR "Stroke Volume"[Mesh] OR "Ventricular Ejection Fraction" OR "Electrocardiography"[Mesh] OR echocardiogram* OR "echo" OR "Heart Rate Determination"[Mesh] OR "cardiac function" OR "heart function" OR "cardiovascular response" OR "circulatory factor*" OR "vascular function" OR "heart rate" OR "stroke volume" OR "cardiac output" OR "aerobic fitness" OR "aerobic capacity" OR "exercise capacity" OR "oxygen pulse" OR "o2 pulse" OR "LVEF" OR "ejection fraction" OR "CPET" OR "Ventilatory threshold") OR ("Respiratory Function Tests"[Mesh] OR "Pulmonary Function" OR "Pulmonary Circulation" OR "Lung Function" OR "Spirometry"[Mesh] OR "Spirometry" OR "Maximal Voluntary Ventilation"[Mesh] OR "MVV" OR "Pulmonary Ventilation"[Mesh] OR "Forced Expiratory Flow Rates"[Mesh] OR "Forced Expiratory Volume"[Mesh] OR "fev1" OR "fvc" OR "ventilation" OR "maximal voluntary ventilation" OR "Exercise Test"[Mesh]) OR ("Pulmonary Gas Exchange"[Mesh] OR "gas exchange" OR "diffusion capacity" OR "blood volume" OR "hemoglobin mass" OR "hb mass" OR "hemoglobin concentration" OR "avo2 difference" OR "arterial oxygen" OR "venous difference" OR "a-v o2 diff" OR "a-vo2" OR "arteriovenous o2 difference" OR "vevco2" OR "ve/vco2" OR "ve vco2" OR "vq mismatch" OR "oxygen saturation" OR "spo2" OR "DLco" OR "Kco" OR "V/Q mismatch" OR "VE/VCO2") OR ("peripheral factor" OR "peripheral factors" OR "circulatory factors" OR "muscle fiber" OR (("muscle") AND ("fiber type" OR "fiber size")) OR "muscular strength" OR "muscle strength" OR "capillarization" OR "capillary density" OR "mitochondrial function" OR "AVO2 difference" OR "skeletal biops*" OR "dissected muscles")) |
| 4 | *Methodological Search Filter |
|  | ((("Clinical Trial"[PT] OR "Comparative Study"[PT] OR "Evaluation study"[PT] OR "Cross-Over Studies"[MeSH] OR "Clinical Trials as Topic"[MeSH] OR random*[TIAB] OR controll*[TIAB] OR "intervention study"[TIAB] OR "experimental study"[TIAB] OR "comparative study"[TIAB] OR trial[TIAB] OR trials[TIAB] OR evaluat*[TIAB] OR repeat*[TIAB] OR compar*[TIAB] OR versus[TIAB] OR "before and after"[TIAB] OR "interrupted time series"[TIAB])) OR ("Epidemiologic Studies"[MeSH] OR "case control"[TIAB] OR "case-control"[TIAB] OR ((case[TIAB] OR cases[TIAB]) AND (control[TIAB] OR controls[TIAB)) OR "cohort study"[TIAB] OR "cohort analysis"[TIAB] OR "follow up study"[TIAB] OR "follow-up study"[TIAB] OR "observational study"[TIAB] OR longitudinal[TIAB] OR retrospective[TIAB] OR "cross sectional"[TIAB] OR questionnaire[TIAB] OR questionnaires[TIAB] OR survey[TIAB])) |
| 5 | Limit: English |
|  | (English[lang]) |
| 6 | Search strategy |
|  | 1 AND 2 AND 3 AND 4 AND 5 |

1. Search strategy translated for **EMBASE (Elsevier)**

|  | Search strategy component concepts |
| --- | --- |
| 1 | Cancer Treatment/Systemic Anticancer Therapy |
|  | (cancer* OR oncolog* OR neoplasm* OR carcinom* OR tumor* OR tumour* OR malignan* OR "hematooncological" OR "hemato-oncological" OR "hematologic neoplasms" OR leukemia*) AND ('antineoplastic agent'/exp OR 'antineoplastic protocol'/exp OR (("Antineoplastic therapy") OR ("cancer” NEXT/3 “therapy") OR (cancer* AND "chemotherapy") OR ("Antineoplastic treatment") OR ("cancer treatment"))) |
| 2 | Cardiorespiratory fitness (CRF) |
|  | ('cardiovascular reserve capacity' OR 'cardiorespiratory capacity' OR 'cardiovascular capacity' OR 'cvrc' OR 'aerobic capacity'/exp OR 'aerobic capacity' OR “aerobic fitness” OR 'vo2' OR 'vo(2)' OR 'peak vo2' OR 'peak vo(2)' OR 'vo2 peak' OR 'vo(2)peak' OR 'vo2peak' OR 'vo(2peak)' OR 'vo 2peak' OR 'peak oxygen consumption' OR 'peak aerobic capacity' OR 'oxygen uptake' OR 'oxygen delivery' OR 'cardiopulmonary function' OR 'ml/kg/min' OR 'oxygen consumption'/exp OR 'oxygen consumption' OR 'max vo2' OR 'max vo(2)' OR 'vo2 max' OR 'vo(2)max' OR 'vo2max' OR 'vo(2max)' OR 'vo 2max' OR 'maximal aerobic capacity' OR 'maximum oxygen consumption' OR 'maximum oxygen uptake' OR 'cardiopulmonary exercise stress test' OR 'cardiopulmonary exercise testing' OR 'cardiopulmonary exercise test' OR 'cpet' OR 'cardiopulmonary exercise test'/exp OR 'exercise test'/exp OR 'exercise test' OR 'exercise testing' OR 'fitness test' OR 'heart function test'/exp OR 'lung function test'/exp OR 'anaerobic threshold'/exp OR 'ventilatory anaerobic threshold' OR “physical fitness” OR 'physical capacity' OR 'physical endurance' OR 'exercise tolerance'/exp OR 'cardiorespiratory fitness' OR 'cardiovascular endurance' OR 'cardiovascular response' OR 'exercise capacity' OR 'heart rate recovery' OR 'hrr') |
| 3 | Physiological determinants of CRF  (Cardiac function/ Pulmonary function/Gas exchange/ Peripheral factors) |
|  | (('heart function test'/exp OR 'heart output'/exp OR 'heart stroke volume'/exp OR "Ventricular Ejection Fraction" OR 'electrocardiography'/exp OR echocardiogram* OR "echo" OR 'heart rate measurement'/exp OR "cardiac function" OR "heart function" OR "cardiovascular response" OR "circulatory factor*" OR "vascular function" OR "heart rate" OR "stroke volume" OR "cardiac output" OR “aerobic fitness” OR “aerobic capacity” OR “exercise capacity” OR "oxygen pulse" OR "o2 pulse" OR "LVEF" OR "ejection fraction" OR "CPET" OR "Ventilatory threshold") OR ('lung function test'/exp OR "Pulmonary Function" OR "Pulmonary Circulation" OR "Lung Function" OR 'spirometry'/exp OR "Spirometry" OR 'maximal voluntary ventilation'/exp OR "MVV" OR 'lung ventilation'/exp OR 'forced expiratory flow'/exp OR 'forced expiratory volume'/exp OR “fev1” OR “fvc” OR "ventilation" OR "maximal voluntary ventilation" OR 'exercise test'/exp) OR ('lung gas exchange'/exp OR "gas exchange" OR "diffusion capacity" OR "blood volume" OR "hemoglobin mass" OR "hb mass" OR "hemoglobin concentration" OR "avo2 difference" OR "arterial oxygen" OR "venous difference" OR "a-v o2 diff" OR "a-vo2" OR "arteriovenous o2 difference" OR "vevco2" OR "ve/vco2" OR "ve vco2" OR "vq mismatch" OR "oxygen saturation" OR "spo2" OR “DLco” OR “Kco” OR "V/Q mismatch" OR “VE/VCO2”) OR ("peripheral factor" OR "peripheral factors" OR "circulatory factors" OR "muscle fiber" OR (("muscle") AND ("fiber type" OR "fiber size")) OR “muscular strength” OR “muscle strength” OR "capillarization" OR "capillary density" OR "mitochondrial function" OR "AVO2 difference" OR "skeletal biops*" OR "dissected muscles")) |
| 4 | *Methodological Search Filter |
|  | ((‘randomized controlled trial’/exp OR ‘clinical trial’/exp OR ‘comparative study’/exp OR ‘controlled study’/de OR ‘evaluation study’/de OR ‘human experiment’/exp OR random*:ab,ti OR control*:ab,ti OR ‘intervention study’:ab,ti OR ‘experimental study’:ab,ti OR ‘comparative study’:ab,ti OR trial:ab,ti OR trials:ab,ti OR compar*:ab,ti OR repeat*:ab,ti OR crossover:ab,ti OR ‘double blind’:ab,ti OR evaluat*:ab,ti OR ‘before and after’:ab,ti OR ‘interrupted time series’:ab,ti) OR (‘clinical study’/exp OR ‘cohort analysis’/exp OR ‘case control’:ab,ti OR ‘case-control’:ab,ti OR ((case:ab,ti OR cases:ab,ti) AND (control:ab,ti OR controls:ab,ti)) OR ‘cohort study’:ab,ti OR ‘cohort analysis’:ab,ti OR ‘follow up study’:ab,ti OR ‘follow-up study’:ab,ti OR ‘observational study’:ab,ti OR longitudinal:ab,ti OR retrospective:ab,ti OR ‘cross sectional’:ab,ti OR questionnaire:ab,ti OR questionnaires:ab,ti OR survey:ab,ti OR ‘epidemiological study’:ab,ti)) |
| 5 | Limit: English |
|  | [english]/lim |
| 6 | Exclude: Publication type – Conference abstracts |
|  | ('conference abstract'/it) |
| 7 | Search strategy |
|  | (1 AND 2 AND 3 AND 4 AND 5) NOT 6 |

(Note: Excluded MEDLINE)

1. Search strategy translated for **CINAHL (EBSCO)**

|  | Search strategy component concepts |
| --- | --- |
| 1 | Cancer Treatment/Systemic Anticancer Therapy |
|  | (cancer* OR oncolog* OR neoplasm* OR carcinom* OR tumor* OR tumour* OR malignan* OR "hematooncological" OR "hemato-oncological" OR "hematologic neoplasms" OR leukemia*) AND ((MH "Antineoplastic Agents+") OR "Antineoplastic Protocols" OR ("Antineoplastic therapy" OR "cancer therapy" OR (cancer* AND "chemotherapy") OR "Antineoplastic treatment" OR "cancer treatment")) |
| 2 | Cardiorespiratory fitness (CRF) |
|  | ("cardiovascular reserve capacity" OR "cardiorespiratory capacity" OR "cardiovascular capacity" OR "CVRC" OR "aerobic capacity" OR “aerobic fitness” OR "VO2" OR "VO(2)" OR "peak VO2" OR "peak VO(2)" OR "Vo2 peak" OR "VO(2)peak" OR "VO2peak" OR "VO(2peak)" OR "VO 2peak" OR "peak oxygen consumption" OR "peak aerobic capacity" OR "oxygen uptake" OR "oxygen delivery" OR "cardiopulmonary function" OR "ml/kg/min" OR "oxygen consumption" OR "max VO2" OR "max VO(2)" OR "VO2 max" OR "VO(2)max" OR "VO2max" OR "VO(2max)" OR "VO 2max" OR "maximal aerobic capacity" OR "maximum oxygen consumption" OR "maximum oxygen uptake" OR "cardiopulmonary exercise stress test" OR "cardiopulmonary exercise testing" OR "cardiopulmonary exercise test" OR "CPET" OR "exercise test" OR "exercise testing" OR "fitness test" OR "Heart Function Tests" OR "Respiratory Function Tests" OR "Anaerobic Threshold" OR "ventilatory anaerobic threshold" OR "Physical Fitness" OR "Physical Endurance" OR "Exercise Tolerance" OR "cardiorespiratory fitness" OR "cardiovascular endurance" OR "cardiovascular response" OR "exercise capacity" OR "heart rate recovery" OR "HRR") |
| 3 | Physiological determinants of CRF  (Cardiac function/ Pulmonary function/Gas exchange/ Peripheral factors) |
|  | ((MH "Heart Function Tests+") OR (MH "Cardiac Output+") OR (MH "Stroke Volume+") OR "Ventricular Ejection Fraction" OR (MH "Electrocardiography+") OR echocardiogram* OR "echo" OR "Heart Rate Determination" OR "cardiac function" OR "heart function" OR "cardiovascular response" OR "circulatory factor*" OR "vascular function" OR "heart rate" OR "stroke volume" OR "cardiac output" OR “aerobic fitness” OR “aerobic capacity” OR “exercise capacity” OR "oxygen pulse" OR "o2 pulse" OR "LVEF" OR "ejection fraction" OR "CPET" OR "Ventilatory threshold") OR ((MH "Respiratory Function Tests+") OR "Pulmonary Function" OR "Pulmonary Circulation" OR "Lung Function" OR (MH "Spirometry") OR "Spirometry" OR "Maximal Voluntary Ventilation" OR "MVV" OR "Pulmonary Ventilation" OR (MH "Forced Expiratory Flow Rates+") OR (MH "Forced Expiratory Volume") OR “fev1” OR “fvc” OR "ventilation" OR "maximal voluntary ventilation" OR (MH "Exercise Test+")) OR ((MH "Pulmonary Gas Exchange+") OR "gas exchange" OR "diffusion capacity" OR "blood volume" OR "hemoglobin mass" OR "hb mass" OR "hemoglobin concentration" OR "avo2 difference" OR "arterial oxygen" OR "venous difference" OR "a-v o2 diff" OR "a-vo2" OR "arteriovenous o2 difference" OR "vevco2" OR "ve/vco2" OR "ve vco2" OR "vq mismatch" OR "oxygen saturation" OR "spo2" OR “DLco” OR “Kco” OR "V/Q mismatch" OR “VE/VCO2”) OR ("peripheral factor" OR "peripheral factors" OR "circulatory factors" OR "muscle fiber" OR (("muscle") AND ("fiber type" OR "fiber size")) OR “muscular strength” OR “muscle strength” OR "capillarization" OR "capillary density" OR "mitochondrial function" OR "AVO2 difference" OR "skeletal biops*" OR "dissected muscles") |
| 4 | *Methodological Search Filter |
|  | ((PT “Clinical Trial”) OR (MH "Comparative Studies+") OR (MH "Clinical Trials+") OR TI (“Evaluation study” OR “Cross-Over Studies” OR random* OR controll* OR “intervention study” OR “experimental study” OR “comparative study” OR trial OR trials OR evaluat* OR repeat* OR compar* OR versus OR “before and after” OR “interrupted time series”) OR AB (“Evaluation study” OR “Cross-Over Studies” OR random* OR controll* OR “intervention study” OR “experimental study” OR “comparative study” OR trial OR trials OR evaluat* OR repeat* OR compar* OR versus OR “before and after” OR “interrupted time series”)) OR (((MH "Epidemiological Research+") OR TI (“case control” OR “case-control”) OR AB (“case control” OR “case-control”) OR ((TI (case OR cases) OR AB (case OR cases)) AND (TI (control OR controls) OR AB (control OR controls))) OR TI (“cohort study” OR “cohort analysis” OR “follow up study” OR “follow-up study” OR “observational study” OR longitudinal OR retrospective OR “cross sectional” OR questionnaire OR questionnaires OR survey) OR AB (“cohort study” OR “cohort analysis” OR “follow up study” OR “follow-up study” OR “observational study” OR longitudinal OR retrospective OR “cross sectional” OR questionnaire OR questionnaires OR survey))) |
| 5 | Limit: English |
|  | Narrow by Language: - english |
| 6 | Search strategy |
|  | 1 AND 2 AND 3 AND 4 AND 5 |

4) Search strategy translated for **SPORTDiscus (EBSCO)**

|  | Search strategy component concepts |
| --- | --- |
| 1 | Cancer Treatment/Systemic Anticancer Therapy |
|  | ((DE "CANCER") OR cancer* OR oncolog* OR neoplasm* OR carcinom* OR tumor* OR tumour* OR malignan* OR "hematooncological" OR "hemato-oncological" OR "hematologic neoplasms" OR leukemia*) AND ((DE "ANTINEOPLASTIC agents") OR (DE "THERAPEUTIC use of antineoplastic agents") OR ("Antineoplastic therapy" OR "cancer therapy" OR (cancer* AND "chemotherapy") OR "Antineoplastic treatment" OR "cancer treatment")) |
| 2 | Cardiorespiratory fitness (CRF) |
|  | ((DE "CARDIOPULMONARY fitness") OR OR (DE "CARDIOPULMONARY fitness measurement") OR "Cardiorespiratory Fitness" OR "cardiovascular reserve capacity" OR "cardiorespiratory capacity" OR "cardiovascular capacity" OR "CVRC" OR "aerobic capacity" OR "aerobic fitness" OR "VO2" OR "VO(2)" OR "peak VO2" OR "peak VO(2)" OR "Vo2 peak" OR "VO(2)peak" OR "VO2peak" OR "VO(2peak)" OR "VO 2peak" OR "peak oxygen consumption" OR "peak aerobic capacity" OR "oxygen uptake" OR "oxygen delivery" OR "cardiopulmonary function" OR "ml/kg/min" OR (DE "OXYGEN consumption") OR "oxygen consumption" OR "max VO2" OR "max VO(2)" OR "VO2 max" OR "VO(2)max" OR "VO2max" OR "VO(2max)" OR "VO 2max" OR "maximal aerobic capacity" OR "maximum oxygen consumption" OR "maximum oxygen uptake" OR "cardiopulmonary exercise stress test" OR "cardiopulmonary exercise testing" OR "cardiopulmonary exercise test" OR "CPET" OR (DE "EXERCISE tests") OR "exercise test" OR "exercise testing" OR "fitness test" OR (DE "HEART function tests") OR (DE "PULMONARY function tests") OR (DE "ANAEROBIC threshold") OR "ventilatory anaerobic threshold" OR (DE "PHYSICAL fitness") OR "physical fitness" OR "physical capacity" OR "Physical Endurance" OR (DE "EXERCISE tolerance") OR "cardiorespiratory fitness" OR "cardiovascular endurance" OR "cardiovascular response" OR "cardiovascular form" OR "exercise capacity" OR "heart rate recovery" OR "HRR") |
| 3 | Physiological determinants of CRF  (Cardiac function/ Pulmonary function/Gas exchange/ Peripheral factors) |
|  | (((DE "HEART function tests") OR (DE "CARDIAC output") OR (DE "STROKE volume (Cardiac output)") OR "Ventricular Ejection Fraction" OR (DE "ELECTROCARDIOGRAPHY") OR echocardiogram* OR "echo" OR (DE "HEART rate monitoring") OR "cardiac function" OR "heart function" OR "cardiovascular response" OR (DE "CARDIOVASCULAR fitness") OR "circulatory factor*" OR "vascular function" OR "heart rate" OR "stroke volume" OR "cardiac output" OR "aerobic fitness" OR "aerobic capacity" OR "exercise capacity" OR "oxygen pulse" OR "o2 pulse" OR "LVEF" OR "ejection fraction" OR "CPET" OR "Ventilatory threshold") OR (OR (DE "PULMONARY function tests") OR "Pulmonary Function" OR "Pulmonary Circulation" OR "Lung Function" OR (DE "SPIROMETRY") OR "Spirometry" OR "Maximal Voluntary Ventilation" OR "MVV" OR "Pulmonary Ventilation" OR "Forced Expiratory Flow Rates" OR "Forced Expiratory Volume" OR "fev1" OR "fvc" OR "ventilation" OR "maximal voluntary ventilation" OR (DE "EXERCISE tests")) OR (OR (DE "PULMONARY gas exchange") OR "gas exchange" OR "diffusion capacity" OR "blood volume" OR "hemoglobin mass" OR "hb mass" OR "hemoglobin concentration" OR "avo2 difference" OR "arterial oxygen" OR "venous difference" OR "a-v o2 diff" OR "a-vo2" OR "arteriovenous o2 difference" OR "vevco2" OR "ve/vco2" OR "ve vco2" OR "vq mismatch" OR "oxygen saturation" OR "spo2" OR "DLco" OR "Kco" OR "V/Q mismatch" OR "VE/VCO2") OR ("peripheral factor" OR "peripheral factors" OR "circulatory factors" OR (DE "MUSCLE strength measurement") OR "muscle fiber" OR (("muscle") AND ("fiber type" OR "fiber size")) OR "muscular strength" OR "muscle strength" OR "capillarization" OR "capillary density" OR "mitochondrial function" OR (DE "MUSCLE mitochondria") OR "AVO2 difference" OR "skeletal biops*" OR "dissected muscles")) |
| 4 | Limit: English |
|  | Narrow by Language: - english |
| 5 | Search strategy |
|  | 1 AND 2 AND 3 AND 4 |

1. Search strategy translated for **Cochrane CENTRAL (Cochrane Library, Wiley)**

|  | Search strategy component concepts |
| --- | --- |
| 1 | Cancer Treatment/Systemic Anticancer Therapy |
|  | (cancer OR oncolog* OR neoplasm* OR carcinom* OR tumor* OR tumour* OR malignan* OR "hematooncological" OR "hemato-oncological" OR "hematologic neoplasms" OR leukemia*) AND ("Antineoplastic Agents"[Mesh] OR "Antineoplastic Combined Chemotherapy Protocols"[Mesh] OR "Antineoplastic Protocols"[Mesh] OR ("Antineoplastic therapy" OR "cancer therapy" OR (cancer* AND "chemotherapy") OR "Antineoplastic treatment" OR "cancer treatment")) |
| 2 | Cardiorespiratory fitness (CRF) |
|  | ("Cardiorespiratory Fitness"[Mesh] OR "Cardiorespiratory Fitness" OR "cardiovascular reserve capacity" OR "cardiorespiratory capacity" OR "cardiovascular capacity" OR "CVRC" OR "aerobic capacity" OR "aerobic fitness" OR "VO2" OR "VO(2)" OR "peak VO2" OR "peak VO(2)" OR "Vo2 peak" OR "VO(2)peak" OR "VO2peak" OR "VO(2peak)" OR "VO 2peak" OR "peak oxygen consumption" OR "peak aerobic capacity" OR "oxygen uptake" OR "oxygen delivery" OR "cardiopulmonary function" OR "ml/kg/min" OR "Oxygen Consumption" OR "oxygen consumption" OR "max VO2" OR "max VO(2)" OR "VO2 max" OR "VO(2)max" OR "VO2max" OR "VO(2max)" OR "VO 2max" OR "maximal aerobic capacity" OR "maximum oxygen consumption" OR "maximum oxygen uptake" OR "cardiopulmonary exercise stress test" OR "cardiopulmonary exercise testing" OR "cardiopulmonary exercise test" OR "CPET" OR "Exercise Test" OR "exercise test" OR "exercise testing" OR "fitness test" OR "Heart Function Tests" OR "Respiratory Function Tests" OR "Anaerobic Threshold" OR "ventilatory anaerobic threshold" OR "Physical Fitness" OR "physical fitness" OR "physical capacity" OR "Physical Endurance" OR "Exercise Tolerance" OR "cardiorespiratory fitness" OR "cardiovascular endurance" OR "cardiovascular response" OR "cardiovascular form" OR "exercise capacity" OR "heart rate recovery" OR "HRR") |
| 3 | Physiological determinants of CRF  (Cardiac function/ Pulmonary function/Gas exchange/ Peripheral factors) |
|  | (("Heart Function Tests" OR "Cardiac Output" OR "Stroke Volume" OR "Ventricular Ejection Fraction" OR "Electrocardiography" OR echocardiogram* OR "echo" OR "Heart Rate Determination" OR "cardiac function" OR "heart function" OR "cardiovascular response" OR "circulatory factor*" OR "vascular function" OR "heart rate" OR "stroke volume" OR "cardiac output" OR "aerobic fitness" OR "aerobic capacity" OR "exercise capacity" OR "oxygen pulse" OR "o2 pulse" OR "LVEF" OR "ejection fraction" OR "CPET" OR "Ventilatory threshold") OR ("Respiratory Function Tests" OR "Pulmonary Function" OR "Pulmonary Circulation" OR "Lung Function" OR "Spirometry" OR "Spirometry" OR "Maximal Voluntary Ventilation" OR "MVV" OR "Pulmonary Ventilation" OR "Forced Expiratory Flow Rates" OR "Forced Expiratory Volume" OR "fev1" OR "fvc" OR "ventilation" OR "maximal voluntary ventilation" OR "Exercise Test") OR ("Pulmonary Gas Exchange" OR "gas exchange" OR "diffusion capacity" OR "blood volume" OR "hemoglobin mass" OR "hb mass" OR "hemoglobin concentration" OR "avo2 difference" OR "arterial oxygen" OR "venous difference" OR "a-v o2 diff" OR "a-vo2" OR "arteriovenous o2 difference" OR "vevco2" OR "ve/vco2" OR "ve vco2" OR "vq mismatch" OR "oxygen saturation" OR "spo2" OR "DLco" OR "Kco" OR "V/Q mismatch" OR "VE/VCO2") OR ("peripheral factor" OR "peripheral factors" OR "circulatory factors" OR "muscle fiber" OR (("muscle") AND ("fiber type" OR "fiber size")) OR "muscular strength" OR "muscle strength" OR "capillarization" OR "capillary density" OR "mitochondrial function" OR "AVO2 difference" OR "skeletal biops*" OR "dissected muscles")) |
| 4 | *Methodological Search Filter |
|  | Limit: in Trials (Cochrane Central Register on Controlled Trials) |
| 5 | Search strategy |
|  | 1 AND 2 AND 3 AND 4 |

(Note: Limit to English**.** Clinical trial registry records excluded.)

***Methodological Search filters consulted/used/adapted:**
Avau B, Van Remoortel H, De Buck E. Translation and validation of PubMed and Embase search filters for identification of systematic reviews, intervention studies, and observational studies in the field of first aid. J Med Libr Assoc. 2021 Oct 1;109(4):599-608. doi: 10.5195/jmla.2021.1219. PMID: 34858089; PMCID: PMC8608173. <https://www.ncbi.nlm.nih.gov/pmc/articles/PMC8608173/>

**Supplemental Appendix 2. PRISMA 2020 for Abstract Checklist**

| **Section and Topic** | **Item #** | **Checklist item** | **Reported (Yes/No)** |
| --- | --- | --- | --- |
| **TITLE** | | |  |
| Title | 1 | Identify the report as a systematic review. | Yes |
| **BACKGROUND** | | |  |
| Objectives | 2 | Provide an explicit statement of the main objective(s) or question(s) the review addresses. | Yes |
| **METHODS** | | |  |
| Eligibility criteria | 3 | Specify the inclusion and exclusion criteria for the review. | Yes |
| Information sources | 4 | Specify the information sources (e.g. databases, registers) used to identify studies and the date when each was last searched. | Yes |
| Risk of bias | 5 | Specify the methods used to assess risk of bias in the included studies. | Yes |
| Synthesis of results | 6 | Specify the methods used to present and synthesise results. | Yes |
| **RESULTS** | | |  |
| Included studies | 7 | Give the total number of included studies and participants and summarise relevant characteristics of studies. | Yes |
| Synthesis of results | 8 | Present results for main outcomes, preferably indicating the number of included studies and participants for each. If meta-analysis was done, report the summary estimate and confidence/credible interval. If comparing groups, indicate the direction of the effect (i.e. which group is favoured). | Yes |
| **DISCUSSION** | | |  |
| Limitations of evidence | 9 | Provide a brief summary of the limitations of the evidence included in the review (e.g. study risk of bias, inconsistency and imprecision). | No |
| Interpretation | 10 | Provide a general interpretation of the results and important implications. | Yes |
| **OTHER** | | |  |
| Funding | 11 | Specify the primary source of funding for the review. | Yes |
| Registration | 12 | Provide the register name and registration number. | No |

*From:* Page MJ, McKenzie JE, Bossuyt PM, Boutron I, Hoffmann TC, Mulrow CD, et al. The PRISMA 2020 statement: an updated guideline for reporting systematic reviews. BMJ 2021;372:n71. doi: 10.1136/bmj.n71

For more information, visit: <http://www.prisma-statement.org/>

**Supplemental Appendix 3. PRISMA 2020 Checklist**

| **Section and Topic** | **Item #** | **Checklist item** | **Location where item is reported** |
| --- | --- | --- | --- |
| **TITLE** | | |  |
| Title | 1 | Identify the report as a systematic review. | Title page |
| **ABSTRACT** | | |  |
| Abstract | 2 | See the PRISMA 2020 for Abstracts checklist. | Title page |
| **INTRODUCTION** | | |  |
| Rationale | 3 | Describe the rationale for the review in the context of existing knowledge. | Introduction |
| Objectives | 4 | Provide an explicit statement of the objective(s) or question(s) the review addresses. | Introduction |
| **METHODS** | | |  |
| Eligibility criteria | 5 | Specify the inclusion and exclusion criteria for the review and how studies were grouped for the syntheses. | Methods, paragraph 2 |
| Information sources | 6 | Specify all databases, registers, websites, organisations, reference lists and other sources searched or consulted to identify studies. Specify the date when each source was last searched or consulted. | Methods, paragraph 1+ supplemental |
| Search strategy | 7 | Present the full search strategies for all databases, registers and websites, including any filters and limits used. | Supplemental |
| Selection process | 8 | Specify the methods used to decide whether a study met the inclusion criteria of the review, including how many reviewers screened each record and each report retrieved, whether they worked independently, and if applicable, details of automation tools used in the process. | Methods, paragraph 3 |
| Data collection process | 9 | Specify the methods used to collect data from reports, including how many reviewers collected data from each report, whether they worked independently, any processes for obtaining or confirming data from study investigators, and if applicable, details of automation tools used in the process. | Methods, paragraph 3 |
| Data items | 10a | List and define all outcomes for which data were sought. Specify whether all results that were compatible with each outcome domain in each study were sought (e.g. for all measures, time points, analyses), and if not, the methods used to decide which results to collect. | Supplemental, table 1 |
|  | 10b | List and define all other variables for which data were sought (e.g. participant and intervention characteristics, funding sources). Describe any assumptions made about any missing or unclear information. | Methods, supplemental |
| Study risk of bias assessment | 11 | Specify the methods used to assess risk of bias in the included studies, including details of the tool(s) used, how many reviewers assessed each study and whether they worked independently, and if applicable, details of automation tools used in the process. | Methods, paragraph 3+ supplemental |
| Effect measures | 12 | Specify for each outcome the effect measure(s) (e.g. risk ratio, mean difference) used in the synthesis or presentation of results. | Methods, paragraph 4 |
| Synthesis methods | 13a | Describe the processes used to decide which studies were eligible for each synthesis (e.g. tabulating the study intervention characteristics and comparing against the planned groups for each synthesis (item #5)). | Methods |
|  | 13b | Describe any methods required to prepare the data for presentation or synthesis, such as handling of missing summary statistics, or data conversions. | Methods, paragraph 4 |
|  | 13c | Describe any methods used to tabulate or visually display results of individual studies and syntheses. | Methods, paragraph 4 |
|  | 13d | Describe any methods used to synthesize results and provide a rationale for the choice(s). If meta-analysis was performed, describe the model(s), method(s) to identify the presence and extent of statistical heterogeneity, and software package(s) used. | Methods, paragraph 4 |
|  | 13e | Describe any methods used to explore possible causes of heterogeneity among study results (e.g. subgroup analysis, meta-regression). | Methods, paragraph 4 |
|  | 13f | Describe any sensitivity analyses conducted to assess robustness of the synthesized results. | NA |
| Reporting bias assessment | 14 | Describe any methods used to assess risk of bias due to missing results in a synthesis (arising from reporting biases). | Methods+ Supplemental |
| Certainty assessment | 15 | Describe any methods used to assess certainty (or confidence) in the body of evidence for an outcome. | NA |
| **RESULTS** | | |  |
| Study selection | 16a | Describe the results of the search and selection process, from the number of records identified in the search to the number of studies included in the review, ideally using a flow diagram. | Results, paragraph 1 + Figure 1 |
|  | 16b | Cite studies that might appear to meet the inclusion criteria, but which were excluded, and explain why they were excluded. | NA |
| Study characteristics | 17 | Cite each included study and present its characteristics. | Supplemental |
| Risk of bias in studies | 18 | Present assessments of risk of bias for each included study. | Results, paragraph 3+ Supplemental |
| Results of individual studies | 19 | For all outcomes, present, for each study: (a) summary statistics for each group (where appropriate) and (b) an effect estimate and its precision (e.g. confidence/credible interval), ideally using structured tables or plots. | Figure 2-4+ Supplemental |
| Results of syntheses | 20a | For each synthesis, briefly summarise the characteristics and risk of bias among contributing studies. | Results, paragraph 2, 3+ Table 2 |
|  | 20b | Present results of all statistical syntheses conducted. If meta-analysis was done, present for each the summary estimate and its precision (e.g. confidence/credible interval) and measures of statistical heterogeneity. If comparing groups, describe the direction of the effect. | Results + Figure 2-5 |
|  | 20c | Present results of all investigations of possible causes of heterogeneity among study results. | Supplement |
|  | 20d | Present results of all sensitivity analyses conducted to assess the robustness of the synthesized results. | NA |
| Reporting biases | 21 | Present assessments of risk of bias due to missing results (arising from reporting biases) for each synthesis assessed. | NA |
| Certainty of evidence | 22 | Present assessments of certainty (or confidence) in the body of evidence for each outcome assessed. | NA |
| **DISCUSSION** | | |  |
| Discussion | 23a | Provide a general interpretation of the results in the context of other evidence. | Discussion |
|  | 23b | Discuss any limitations of the evidence included in the review. | Discussion, paragraph “limitations” |
|  | 23c | Discuss any limitations of the review processes used. | Discussion, paragraph “limitations” |
|  | 23d | Discuss implications of the results for practice, policy, and future research. | Discussion |
| **OTHER INFORMATION** | | |  |
| Registration and protocol | 24a | Provide registration information for the review, including register name and registration number, or state that the review was not registered. | Methods, paragraph 2 |
|  | 24b | Indicate where the review protocol can be accessed, or state that a protocol was not prepared. | Methods, paragraph 1 |
|  | 24c | Describe and explain any amendments to information provided at registration or in the protocol. | NA |
| Support | 25 | Describe sources of financial or non-financial support for the review, and the role of the funders or sponsors in the review. | Title page |
| Competing interests | 26 | Declare any competing interests of review authors. | Title page |
| Availability of data, code and other materials | 27 | Report which of the following are publicly available and where they can be found: template data collection forms; data extracted from included studies; data used for all analyses; analytic code; any other materials used in the review. | NA |

*From:*  Page MJ, McKenzie JE, Bossuyt PM, Boutron I, Hoffmann TC, Mulrow CD, et al. The PRISMA 2020 statement: an updated guideline for reporting systematic reviews. BMJ 2021;372:n71. doi: 10.1136/bmj.n71

For more information, visit: <http://www.prisma-statement.org/>

**Supplemental Appendix 4. Endpoints and extracted variables**

**Supplemental Table 1.** Endpoint variables of interest and associated measurement methods.

| **CRF** | **Determinants of CRF** | **Measurement** |
| --- | --- | --- |
| VO_2peak_ | Cardiovascular function  Cardiac output  Stroke volume | Stress echocardiography |
|  | Pulmonary function  FEV_1_  FVC | Spirometry/flow-volume |
|  | Gas exchange  DLco  Hemoglobin mass  Blood volume | Diffusion capacity for carbon monoxide  and/or test of hemoglobin mass |
|  | Peripheral function  Muscle fiber type  Muscle fiber size  Capillarization  Mitochondrial function  a-vO_2_ difference | Skeletal biopsies  Directly measured or indicated |

Cardiorespiratory fitness (CRF), peak oxygen consumption (VO_2peak_), Forced Expiratory Volume in the first second (FEV_1_), Forced Vital Capacity (FVC), Diffusion Capacity for Carbon Monoxide in the lung (DLco), arteriovenous oxygen difference (a-vO_2_ difference).

The following study details were extracted:

*Study characteristics* (title, first author, year of publication, country, study design),

*Study population* (cancer site, age, BMI, gender, time since cancer onset, total number of participants, loss to follow up)

*Treatment characteristics* (treatment setting, cancer treatment type, time of treatment)

*Cardiorespiratory fitness assessment* (modality, protocol, measurement system)

*Assessment for determinants* (methods for pulmonary function/cardiac function/avO_2_difference/muscle biopsy)

*Endpoint variables* (VO_2peak_ in mL·kg^-1^·min^-1^, peak cardiac output, peak stroke volume and avO_2_difference).

**Supplemental Appendix 5. Quality assessment**

Risk of bias in the included studies was assessed according to the respective study design. For randomized controlled trials, the Cochrane Risk of Bias Tool was used, which comprises the following six domains: (1) Sequence generation, (2) allocation concealment, (3) blinding, (4) incomplete data outcome, (5) selective outcome reporting and (6) other potential threats to validity^1^. Each domain was categorized as either (+) yes, (-) no, or (?) unclear. Similarly, the “NIH Quality Assessment Tool for Observational Cohort and Cross-Sectional Studies” and the “NIH Quality Assessment Tool for Before-After Studies With no Control Group” were used for cross-sectional- and prospective trials, respectively^2^.

**Supplemental Table 2.** Cochrane Risk of Bias Tool for clinical trials.

| **Trial** | **Was the allocation sequence adequately generated? (Selection bias)** | **Was allocation adequately concealed? (Selection bias)** | **Blinding (Performance bias)** | **Were incomplete outcome data adequately addressed? (Attrition bias)** | **Are reports of the study free of suggestion of selective outcome reporting? (Reporting bias)** | **Was the study apparently free of other problems that could put it at a risk of bias? (Other bias)** |
| --- | --- | --- | --- | --- | --- | --- |
| West et al., 2015^3^ | No | No | NA | Yes | Yes | No |
| Mowafy et al., 2016^4^ | No | No | NA | No | No | No |
| Egegaard et al., 2019^5^ | Yes | Yes | NA | No | Yes | No |
| Quist et al., 2020^6^ | Unclear | Yes | NA | Yes | Yes | Yes |
| van der Schoot et al., 2022^7^ | Yes | Yes | NA | Yes | Yes | Yes |
| Courneya et al., 2007^8^ | Yes | Yes | NA | Yes | Yes | Yes |
| Vincent et al., 2020^9^ | Unclear | Unclear | NA | Yes | Unclear | Yes |
| Howden et al., 2019^10^ | No | No | NA | Yes | No | No |
| Cornette et al., 2016^11^ | Unclear | Unclear | NA | Yes | Yes | Yes |
| Ndjavera et al., 2020^12^ | Yes | Yes | NA | Yes | Yes | Yes |
| Al-Majid et al., 2015^13^ | Unclear | Unclear | NA | Unclear | Yes | Yes |
| Allen et al., 2022^14^ | Yes | Unclear | NA | No | Yes | Yes |
| Chung et al., 2022^15^ | Yes | Yes | NA | Yes | Yes | Yes |
| Hornsby et al., 2014^16^ | Yes | Yes | NA | Yes | Yes | Yes |
| Foulkes et al., 2023^17^ | Yes | Yes | NA | Yes | Yes | Yes |
| Schneider et al., 2023^18^ | Yes | Yes | NA | Unclear | Yes | Yes |
| Scott et al., 2023^19^ | Yes | Yes | NA | Yes | Yes | Yes |

Abbreviation: NA (not applicable)

**Supplemental Table 3.** NIH Quality Assessment Tool for Prospective Cohort Studies

|  | **Was the research question or objective clearly stated? (Q1)** | **Were eligibility/selection criteria for the study population prespecified and clearly described? (Q2)** | **Were the participants in the study representative of those who would be eligible for the test/service/intervention in the general or clinical population of interest? (Q3)** | **Were all eligible participants that met the prespecified entry criteria enrolled? (Q4)** | **Was the sample size sufficiently large to provide confidence in the findings? (Q5)** | **Was the test/service/intervention clearly described and delivered consistently across the study population? (Q6)** | **Were the outcome measures prespecified, clearly defined, valid, reliable, and assessed consistently across all study participants? (Q7)** | **Were the people assessing the outcomes blinded to the participants' exposures/interventions? (Q8)** | **Was the loss to follow-up after baseline 20% or less? Were those lost to follow-up accounted for in the analysis? (Q9)** | **Did the statistical methods examine changes in outcome measures from before to after the intervention? Were statistical tests done that provided p values for the pre-to-post changes? (Q10)** | **Were outcome measures of interest taken multiple times before the intervention and multiple times after the intervention (i.e., did they use an interrupted time-series design)? (Q11)** | **If the intervention was conducted at a group level (e.g., a whole hospital, a community, etc.) did the statistical analysis take into account the use of individual-level data to determine effects at the group level? (Q12)** |
| --- | --- | --- | --- | --- | --- | --- | --- | --- | --- | --- | --- | --- |
| Kirkham et al., 2022^20^ | Yes | Yes | Yes | Yes | Yes | Yes | Yes | Other* | Other* | Yes | No | Other* |
| West et al., 2021^21^ | Yes | Yes | Yes | Yes | Other* | Yes | Yes | Yes | No | Yes | No | Other* |
| Thomson et al., 2018^22^ | Yes | Yes | Yes | Yes | Yes | Yes | Yes | Other* | Yes | Yes | No | Other* |
| Jack et al., 2014^23^ | Yes | Yes | Yes | Yes | Yes | Yes | Yes | Yes | No | Yes | No | Other* |
| Navidi et al., 2018^24^ | Yes | Yes | Yes | Yes | No | Yes | Yes | Yes | No | Yes | No | Other* |
| Groehs et al., 2020^25^ | Yes | Yes | Yes | Yes | Yes | Yes | Yes | Other* | Other* | Yes | No | Other* |
| Culos-Reed et al., 2017^26^ | Yes | No | Yes | Yes | No | Yes | Yes | Other* | No | Yes | No | Other* |
| Wiestad et al., 2020^27^ | Yes | Yes | Yes | Yes | Other* | Yes | Yes | Other* | No | Yes | No | Other* |
| Howden et al., 2021^28^ | Yes | Yes | Yes | Yes | No | Yes | Yes | Other* | Yes | Yes | No | Other* |
| Dillon et al., 2022^29^ | Yes | Yes | Yes | Yes | Yes | Yes | Yes | Other* | Yes | Yes | No | Other* |

*Cannot determine/not applicable/not reported

**Table 4.** NIH Quality Assessment Tool for Cross-sectional Studies

|  | **Was the research question or objective in this paper clearly stated? (Q1)** | **Was the study population clearly specified and defined? (Q2)** | **Was the participation rate of eligible persons at least 50%? (Q3)** | **Were all the subjects selected or recruited from the same or similar populations (including the same time period)? Were inclusion and exclusion criteria for being in the study prespecified and applied uniformly to all participants? (Q4)** | **Was a sample size justification, power description, or variance and effect estimates provided? (Q5)** | **For the analyses in this paper, were the exposure(s) of interest measured prior to the outcome(s) being measured? (Q6)** | **Was the timeframe sufficient so that one could reasonably expect to see an association between exposure and outcome if it existed? (Q7)** | **For exposures that can vary in amount or level, did the study examine different levels of the exposure as related to the outcome (e.g., categories of exposure, or exposure measured as continuous variable)? (Q8)** | **Were the exposure measures (independent variables) clearly defined, valid, reliable, and implemented consistently across all study participants? (Q9)** | **Was the exposure(s) assessed more than once over time? (Q10)** | **Were the outcome measures (dependent variables) clearly defined, valid, reliable, and implemented consistently across all study participants? (Q11)** | **Were the outcome assessors blinded to the exposure status of participants? (Q12)** | **Was loss to follow-up after baseline 20% or less? (Q13)** | **Were key potential confounding variables measured and adjusted statistically for their impact on the relationship between exposure(s) and outcome(s)? (Q14)** |
| --- | --- | --- | --- | --- | --- | --- | --- | --- | --- | --- | --- | --- | --- | --- |
| Baracos et al., 1994^30^ | Yes | No | Other* | Other* | No | No | Yes | No | Yes | No | Yes | No | Other* | No |
| Koelwyn et al., 2016^31^ | Yes | Yes | No | Yes | Yes | No | Yes | No | Yes | No | Yes | No | Other* | Yes |
| Beaudry et al., 2020^32^ | Yes | No | Other* | Yes | No | No | Yes | No | Yes | No | Yes | No | Other* | Yes |
| Kirkham et al., 2013^33^ | Yes | No | Other* | No | No | No | Yes | No | Yes | No | Yes | No | Other* | No |
| Jones et al., 2007^34^ | Yes | No | Yes | Yes | No | No | Yes | Yes | Yes | No | No | Yes | Other* | Yes |
| Crowgey et al., 2014^35^ | Yes | Yes | Other* | Yes | No | No | Yes | No | No | No | No | No | Other* | No |
| Khouri et al., 2014^36^ | Yes | Yes | Other* | Yes | No | No | Yes | Yes | Yes | No | Yes | Yes | Other* | Yes |
| Beaudry et al., 2022^37^ | Yes | No | Other* | Other* | No | No | Yes | No | No | No | Yes | No | Other* | No |
| Kirkham et al., 2021^38^ | Yes | Yes | No | Yes | No | No | Yes | Yes | Yes | No | Yes | No | Other* | Yes |
| Jones et al., 2007^39^ | Yes | No | No | No | No | No | Yes | No | Yes | No | Yes | No | Other* | No |
| Yu et al., 2020^40^ | Yes | Yes | Other* | Yes | Yes | No | Yes | Yes | Yes | No | Yes | No | Other* | Yes |
| Long et al., 2020^41^ | Yes | Yes | Other* | Yes | Yes | No | Yes | No | Yes | No | Yes | No | Other* | No |
| Cramer et al., 2014^42^ | No | Yes | Other* | Yes | No | No | Other* | No | No | No | Yes | No | Other* | No |
| Reding et al., 2019^43^ | No | No | Other* | Yes | No | No | Yes | Yes | Yes | No | No | Yes | Other* | Yes |
| Tonorezoz et al., 2013^44^ | Yes | Yes | Yes | Yes | No | Yes | Yes | Yes | Yes | No | Yes | Other* | Other* | Yes |
| Ness et al., 2020^45^ | Yes | Yes | Yes | Yes | No | No | Yes | Yes | Yes | No | Yes | No | Other* | Yes |
| Caru et al., 2019^46^ | Yes | Yes | Other* | Yes | No | No | Yes | No | Yes | No | Yes | No | Other* | Yes |

*Cannot determine/not applicable/not reported NA

**S6. Funnel plots**


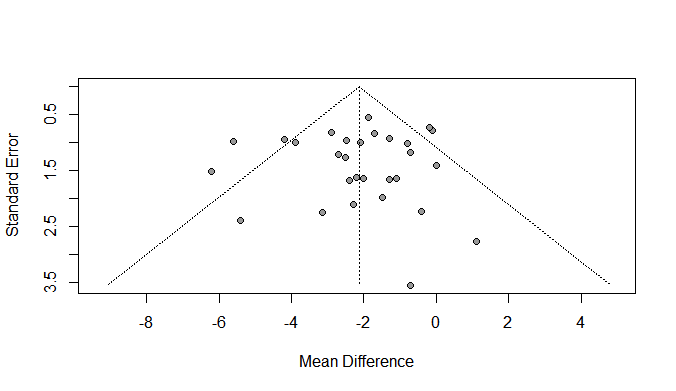


**Supplemental Figure 1.** Funnel plot of the clinical trials.


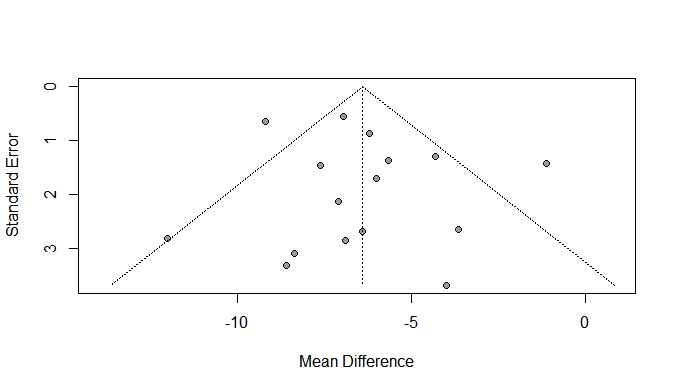


**Supplemental Figure 2.** Funnel plot of the cross-sectional studies.

**S7. Descriptive study characteristics**

**Supplemental Table 5**. Descriptive study- and treatment characteristics for the included Randomized Controlled Trials and Prospective Cohort Studies.

| **Study** | **Study design** | **Cancer Site** | **Age at study** | **Timepoint for post assessment** | **Cancer treatment** |
| --- | --- | --- | --- | --- | --- |
| Allen et al., 2022^14^ | RCT | Oseophago-gastric | 629 | 2 weeks post treatment | Neoadjuvant chemotherapy (n=27, 96%) or chemoradiation (n=1, 4%) |
| Al-Majid et al., 2015^13^ | RCT | Breast | 52.710.7 | At the completion of chemotherapy | *Chemotherapy* |
| Chung et al., 2022^15^ | RCT | Breast | 50.37.7 | At completion of chemotherapy | *Chemotherapy*  CEF (Cyclophosphamide 500 mg/m^2^+ Epirubicin 75 mg/m^2^+ 5-FU 500 mg/m^2^): 54%. AC followed by T (Doxorubicin 60 mg/m^2^+ cyclophosphamide 600 mg/m^2^+ docetaxel 60 mg/m^2^): 46% |
| Cornette et al., 2016^11^ | RCT | Breast | 49 (37-68) | At completion + 6.5 months post-treatment | *Chemotherapy*  3 courses FEC100+ 3 courses Taxotere |
| Courneya at al., 2007^8^ | RCT | Breast | 49 (26-78) | 3-4 weeks post treatment | *Adjuvant chemotherapy*  Non-Taxane (65.4%): FEC100c, AC, CE120F or other. Taxane (34.1%): TAC, AC-Taxane or other |
| Culos-Reed et al., 2017^26^ | Prospective | Head-neck/CNS | 50.63.7 | Post treatment+ 6 months post treatment | *Chemoradiotherapy*  Temozolomide. N=15, 100% |
| Dillon et al., 2022^29^ | Prospective | Leukemia/  lymphoma | 4518 | 3 months following Allogeneic hematopoietic cell transplantation | *Allogeneic hematopoietic cell transplantation*  Graft source: Bone marrow (n=3, 18%), peripheral blood stem cell (n=14, 82%). Previous chemotherapy: 94% (Anthracyclines and anti-metabolite most frequently administered). |
| Egegaard et al., 2019^5^ | RCT | Lung | 654.7 | 0-9 days after last fraction | *Chemoradiation*  Cisplatin+ Vinorelbine+ radiation |
| Foulkes et al., 2023^17^ | RCT | Breast | 51.27.6 | 4 weeks post completion of AC | *Chemotherapy*  4 cycles of 60 mg/m^2^ doxorubicin combined with 600 (n=50, 100%). mg/m^2^ cyclophosphamide.  Additional taxane: n=46 (92%)  Left-sided radiation: n=18 (36%)  HER2-targeted therapy: n=12 (24%)  Aromatase inhibitor: n=20 (40%)  Immunotherapy: n=3 (6%) |
| Groehs et al., 2015^25^ | Prospective | Colon/rectum | 601 | 2 weeks post chemotherapy | *Adjuvant chemotherapy*  5-fluorouracil (370 mg/m^2^) + Leucovorin 50/mg^2^ weekly for 24-30 weeks: n=12, 41%. 5-Fluorouracil (500 mg/m^2^) + Leucovorin (20 mg/m^2^) + Oxaliplatin (85 mg/m^2^) for 24 weeks: n=17, 59% |
| Hornsby et al., 2014^16^ | RCT | Breast | 4611 | At the completion of chemotherapy | *Chemotherapy*  Doxorubicin (60 mg/m^2^) + Cyclophosphamide (600 mg/m^2^) |
| Howden et al., 2019^10^ | Non-RCT | Breast | 5212 | 3 weeks post treatment | *Chemotherapy*  Anthracycline (Doxorubicin/ Cyclophosphamide): n=11, 79%.  Anthracycline (dose/dense): n=2, 14%. FEC-D: n=1, 7% |
| Howden et al., 2021^28^ | Prospective | Mix | 5313 | ﻿3 months post treatment | *Chemotherapy; allogenic SCT tyrosine kinase; endocrine agents*  Chemotherapy (n=109, 53%). ADT (n=19, 10%). Bruton’s Tyrosine kinase inhibitor (n=39, 19%). Allogenic transplantation (n=39, 19%). |
| Jack et al., 2014^23^ | Prospective | Oseophago-gastric | 64.869.06 | 4 weeks post treatment | *Neoadjuvant chemotherapy*  NR |
| Kirkham et al., 2022^20^ | Prospective | Breast | 5110 | At the end of chemotherapy+ 6 months post chemo | *Chemotherapy; targeted/biological agents; radiation*  Anthracycline containing (Epirubicin, combined with Fluorouracil and Cyclophosphamide, followed by Docetaxel): N=24, 71%. Trastuzumab containing + Docetaxel and Carboplatin: N=10, 29%. Total radiation: n=31, 91%. Radiation left sided: n=18, 53% |
| Mowafy et al., 2016^4^ | Non-RCT | Breast | 35-55 | At the end of 4 months of treatment | *Chemotherapy* |
| Navidi et al., 2018^24^ | Prospective | Oseophago-gastric | 65 (41-81) | 7 days post treatment | *Neoadjuvant chemotherapy*  ECX-regimen (Epirubicin 50 mg/m^2^, Cisplatin 60 mg/m^2^, Capecitabine 652 mg/m^2^): n=31, 100% |
| Ndjavera et al., 2020^12^ | RCT | Prostate | 72.54.2 | ﻿After 3 and 6 months of ADT | *Endocrine agents*  Androgen-deprivation therapy |
| Quist et al., 2020^6^ | RCT | Lung | 63.58.7 | ﻿NR | *Chemoradiation*  Non-Taxane; Carboplatin & Vinorelbine; Carboplatin & Bevacizumab & Vinorelbine or Carboplatin & Etoposide or Cisplatin & Vinorelbine or Cisplatin & Etoposide or Cisplatin & Topotecan or Pemetrexed or Erlotinib or Crizotinib (n=105, 97.2%). Docetaxel (n= 3, 2.8%). Radiotherapy (n=74, 68.5%) |
| Schneider et al., 2023^18^ | RCT | Mix | 46 (38-57) | ﻿In the first 2 weeks upon AC completion | *Combined: Chemotherapy; targeted/biological agents; radiation; endocrine agents*  Breast cancer: Anthracyclines Lymphoma: BEACOPP or ABVD with Doxorubicin. Taxol (n=38, 66.7%). Radiation (n=20, 69%). Hormone therapy (n=7, 24.1%). Herceptin (n=7, 24.1%) |
| Scott et al., 2023^19^ | RCT | Breast | 4510 | At completion of the final chemotherapy cycle | *Neoadjuvant& adjuvant*  *Chemotherapy; radiation; endocrine agents; targeted/biological agents;*  Anthracyclines (n=33, 85%). Anthracycline and Capecitabine (n=1, 3%). Radiotherapy (n=32, 82%). Endocrine therapy (n=28, 72%). Antibody therapy (n=9, 23%) |
| Thomson et al., 2018^22^ | Prospective | Oseophago-gastric | 6610.5 | 4 weeks post treatment | *Neoadjuvant chemotherapy& chemoradiation*  Cisplatin+ 5-fluorouracil (n=13, 33%). Cisplatin+ 5-fluorouracil+ docetaxel (n=7, 18%). Cisplatin+ 5-fluorouracil+ Docetaxel+ Epirubicin (n=2, 5%). Radiation (n=16, 42%) |
| van der Schoot et al., 2022^7^ | RCT | Mix; testicular; breast; colon/rectum | 48.3 (21-76) | Immediately post chemotherapy | *Adjuvant chemotherapy; targeted/biological agents*  Testicular: BEP/EP (n=43, 91%). Other (n=4, 9%). Breast:  Anthracyclines (n=58, 84%). Chemotherapy without Anthracyclines (n=11, 16%). Additional Trastuzumab (n=16, 23%). Colon: FOLFOX (n=12, 75%). CAPOX (n=1, 6%). FOLFOX+CAPOX (n=3, 19%) |
| Vincent et al., 2020^9^ | RCT | Breast | 50 (37-72) | 6 months post treatment | *Chemotherapy*  3+3 courses FEC100+ Docetaxel |
| West et al., 2015^3^ | Non-RCT | Colon/rectum | 72 (62-84) | Immediately post chemotherapy | *Neoadjuvant chemoradiotherapy*  Capecitabine (825 mg/m2) |
| West et al., 2021^21^ | Prospective | Oseophago-gastric | 67 (55-79) | Approx. 4 weeks post chemotherapy | *Neoadjuvant chemotherapy*  Only chemotherapy (n=102, 75%). Chemoradiation (n=34, 75%). Chemotherapy regimen: Epirubicin, Oxaliplatin, Capecitabine (EOX) or Epirubicin, Cisplatin, Capecitabine (ECX) or Epirubicin, Cisplatin, 5‐fluorouracil (ECF) or ECX or ECX + Bevacizumab or CX or Cisplatin and 5‐fluorouracil |
| Wiestad et al., 2020^27^ | Prospective | Mix; breast; colon/rectum | 58.811.1 | 6 months after start of adjuvant treatment | *Chemotherapy; endocrine agents*  Chemotherapy group: Docetaxel 75-80 mg/m^2^+ FEC low-dose 75-100 mg/m^2^ (n=9, 45%). Docetaxel 90-100 mg/m^2^+ FEC high dose (n=8, 40%). Capecitabine and Oxaliplatin (n=1, 5%). Capecitabine single (n=1, 5%). Endocrine therapy (n=17) |

Data presented as mean or median (range). Abbreviations: Central nervous system (CNS), Anthracyclines (AC), Androgen deprivation therapy (ADT), not reported (NR), randomized controlled trial (RCT), Stem cell transplantation (SCT)

**Supplemental Table 6.** Descriptive study- and treatment characteristics for the included Cross-Sectional Studies.

| **Study** | **Design** | **Cancer site** | **Age** | **Time since diagnosis/ treatment administered** | **Cancer treatment** |
| --- | --- | --- | --- | --- | --- |
| Baracos et al., 1994^30^ | Cross-sectional | Lung | CS: 55 (SEM 3)  HC: 53 (SEM 3) | NR/6 weeks post treatment | *Chemoradiation*  Alternating VP-16, CDDP (Etoposide 100 mg/m^2^, Cisplatinum 5 mg/m^2^) and VAC (Vincristine, Doxorubicin 50 mg/m^2^, Cyclophosphamide 100 mg/m^2^) (n=11, 100%). Thoracic irradiation (n=11, 100%) |
| Beaudry et al., 2020^32^ | Cross-sectional | Breast | CS: 5610  HC: 5610 | ﻿NR/12.8 months after administration of the final anthracycline treatment | *Chemotherapy*  Epirubicin/Doxorubicin (n=16, 100%) |
| Beaudry et al., 2022^37^ | Cross-sectional | Breast | CS: 673  HC: 675 | NR/9.8 years post treatment with anthracyclines | *Chemotherapy*  Anthracyclines (n=9, 100%) |
| Caru et al., 2019^46^ | Cross-sectional | Leukemia | CS: 21.86.4  HC: 23.911.0 | NR/Diagnosed between 1987 and 2010 and were <19 years at diagnosis | *Chemotherapy* |
| Cramer et al., 2014^42^ | Cross-sectional | Colon/  rectum | CS:59.912.0  HC: 61.311.2 | ﻿22 months since diagnosis/NR | *Chemotherapy*  5-fluorouracil (n=21, 81%), Capecitabine (n=2, 8%), Irinotecan (n=10, 38%), Bevacizumab (n=11, 42%), Cetuximab/Panitumumab/  Mitomycin (n=5, 19%) |
| Crowgey et al., 2014^35^ | Cross-sectional | Breast | CS: 5212  HC: 585 | 21.7 months/16.3 months since chemotherapy | *Chemotherapy*  Doxorubicin (n=37, 100%). Additional cytotoxic therapy (Taxol, Xeloda, Abraxane): n=31, 84% |
| Jones et al., 2007^34^ | Cross-sectional | Breast | CS: 488.5  HC: 45.28.3 | 30 months/20 months since chemotherapy completion | *Chemotherapy; targeted/biological agents radiation; endocrine therapy.*  THC: Six cycles of concurrent Docetaxel (75 mg/m^2^) with platinum and concurrent Trastuzumab for 1 year (n=11, 42%). AC-TH: Four cycles of Doxorubicin (60 mg/m^2^) and Cyclophosphamide (600 mg/m^2^) followed by four cycles of Docetaxel (100 mg/m^2^) with concurrent Trastuzumab for 1 year (n=8, 31%). AC-T: Four cycles of Doxorubicin (60 mg/m^2^) and Cyclophosphamide (600 mg/m^2^) followed by four cycles of Docetaxel (100 mg/m^2^) (n=7, 27%). Radiation: 65%. Tamoxifen: 62%. Trastuzumab (total for both groups): n=18, 69% |
| Jones et al., 2007^39^ | Cross-sectional | Breast | CS: 597  HC: 565 | 38 months/34 months since chemotherapy | *Adjuvant chemotherapy; radiotherapy; endocrine therapy*  Anthracyclines (n=47, 100%). Left-sided radiation (28%).  Tamoxifen (n=26, 55%). Aromatase inhibitor (n=21, 45%) |
| Khouri et al., 2014^36^ | Cross-sectional | Breast | CS: 5110  HC: 577 | ﻿30 months/26 months post chemotherapy completion | *Adjuvant chemotherapy; radiation; endocrine therapy*  Doxorubicin (n=57). Epirubicin (n=1). Additional cytotoxic therapy (Taxol, Xeloda, and Abraxane): n=47, 82%. Additional radiation (n=45, 79%). Endocrine therapy (n=44, 77%) |
| Kirkham et al., 2013^33^ | Cross-sectional | Breast | Patients: 47.73.7  Survivors: 525.7  HC: 54.34.5 | ﻿NR/Patients: 3.5 weeks since chemotherapy. Survivors: 87,5 weeks since chemotherapy | *Chemotherapy; radiation; endocrine therapy.*  Patients: Doxorubicin+ Cyclophosphamide (n=4, 40%).  Docetaxel+ Cyclophosphamide (n=2, 20%) FECD or FECDT (n=4, 40%). Survivors: Doxorubicin+ Cyclophosphamide: (n=3, 30%). Docetaxel+ Cyclophosphamide (n=1, 10%). FECD or FECDT (n=6, 60%) |
| Kirkham et al., 2021^38^ | Cross-sectional | Breast | CS: 5610  HC: 5610 | NR/Mean time 12.8 months since ended treatment | *Adjuvant chemotherapy; radiation; endocrine therapy*  3× Fluorouracil, Epirubicin & Cyclophosphamide+ 3× Docetaxel (n=14, 88%). 6× Fluorouracil, Epirubicin & Cyclophosphamide (n=1, 6%). 5× Fluorouracil, Doxorubicin & Cyclophosphamide (n=1, 6%). Capecitabine (n=2, 13%). External beam (n=15, 94%).  Current aromatase inhibitor (n=5, 31%). Current tamoxifen (n=4, 25%). Both (n=2). |
| Koelwyn et al., 2016^31^ | Cross-sectional | Breast | CS: 617  HC: 628 | ﻿7 years/Mean time 6.5 years since completion of Anthracyclines treatment | *Chemotherapy; radiation; endocrine therapy.* Adjuvant  Anthracyclines (Doxorubicin or Epirubicin or combination: N=33, 100%. Taxol: n=10, 33%. Radiation: n=23, 77%. Endocrine therapy: n=30, 100%. Tamoxifen: n=7, 23%. Aromatase inhibitor: n=8, 27%. Both: n=15, 50% |
| Long et al., 2020^41^ | Cross-sectional | Leukemia | CS: 193  HC: 222 | ﻿12 years/Mean time since final treatment was 12 ± 4 years | *Chemotherapy; Hematopoietic stem cell transplantation*  Anthracyclines (n=19, 100%). Cyclophosphamide (n=15, 79%). HSCT (n=3). |
| Ness et al., 2020^45^ | Cross-sectional | Mix childhood cancers | CS: 35.78.2  HC: 34.510 | NR/26.2 years since diagnosis | *Chemotherapy; radiation*  Anthracyclines (n=526, 79%). Alkylating agents (n=477, 71.6%). Bleomycin (n=n=60, 9%). Cisplatin (n=72, 10.8%), Carboplatin (n=48, 7.2%). Vincristine (n=470, 70.6%). Methotrexate (n=130 19.5%). |
| Reding et al., 2019^43^ | Cross-sectional | Breast & lymphoma | CS: 5417  HC: 5415 | NR/>12 months post-cancer | *Chemotherapy*  Doxorubicin (n=14, 100%) |
| Tonorezos et al., 2013^44^ | Cross-sectional | Leukemia | CS: 24.34.9  HC: NR | ﻿NR/Survivors were many years from cancer treatment; greater than two thirds were 15 or more years from therapy | *Chemotherapy; Radiotherapy*  Anthracyclines (n=83, 72%). Dexamethasone (n= 12, 10.4%). Cyclophosphamide (n=47, 40.9%). Cranial radiotherapy (n=39, 34%). |
| Yu et al., 2020^40^ | Cross-sectional | Breast | CS: 60.8  HC: 60.9 | ﻿NR/Median time since completion of targeted therapy for ERBB2-positive cancer was 7.0 (IQR, 6.2-8.7) years | *Adjuvant chemotherapy; targeted/biological agents; radiotherapy*.  Anthracycline-based  TOX-group (n=20, 91%), NOTOX-group (n=18, 90%).  No anthracycline-based  TOX-group (n=2, 9%),  NOTOX-group (n=2, 10%). Radiation: TOX-group (n=13, 59%), NOTOX-group (n=12, 60%). Trastuzumab:TOX-group (n=20, 91%), NOTOX-group (n=20, 100%). Trastuzumab+Pertuzumab TOX-group (n=2, 9%). |

Data presented as mean or median (range). Abbreviations: Cancer survivors (CS), Healthy controls (HC), Not reported (NR), Hematopoietic stem cell transplantation (HSCT)

References

1. Higgins JPT, Altman DG, Gøtzsche PC, Jüni P, Moher D, Oxman AD, mfl. The Cochrane Collaboration’s tool for assessing risk of bias in randomised trials. BMJ. 18. oktober 2011;343:d5928.

2. National Heart Lung and Blood Institute. Study Quality Assessment Tools. 2021; Tilgjengelig på: https://www.nhlbi.nih.gov/health-topics/study-quality-assessment-tools

3. West MA, Loughney L, Lythgoe D, Barben CP, Sripadam R, Kemp GJ, mfl. Effect of prehabilitation on objectively measured physical fitness after neoadjuvant treatment in preoperative rectal cancer patients: a blinded interventional pilot study. Br J Anaesth. 2015;114(2):244–51.

4. Mowafy ZME, Zoheiry IMI, Elmonem MGA, Katter D. Efficacy of aerobic training on maximal oxygen consumption and total leukocytes count after chemotherapy in breast cancer patients. International Journal of PharmTech Research. 2016;9(4):34–40.

5. Egegaard T, Rohold J, Lillelund C, Persson G, Quist M. Pre-radiotherapy daily exercise training in non-small cell lung cancer: A feasibility study. Reports of Practical Oncology & Radiotherapy. 2019;24(4):375–82.

6. Quist M, Langer SW, Lillelund C, Winther L, Laursen JH, Christensen KB, mfl. Effects of an exercise intervention for patients with advanced inoperable lung cancer undergoing chemotherapy: A randomized clinical trial. Lung Cancer. 2020;145:76–82.

7. van der Schoot GGF, Ormel HL, Westerink NDL, May AM, Elias SG, Hummel YM, mfl. Optimal Timing of a Physical Exercise Intervention to Improve Cardiorespiratory Fitness. JACC CardioOncol. 2022;4(4):491–503.

8. Courneya KS, Segal RJ, Mackey JR, Gelmon K, Reid RD, Friedenreich CM, mfl. Effects of aerobic and resistance exercise in breast cancer patients receiving adjuvant chemotherapy: a multicenter randomized controlled trial. J Clin Oncol. 2007;25(28):4396–404.

9. Vincent F, Deluche E, Bonis J, Leobon S, Antonini MT, Laval C, mfl. Home-Based Physical Activity in Patients With Breast Cancer: During and/or After Chemotherapy? Impact on Cardiorespiratory Fitness. A 3-Arm Randomized Controlled Trial (APAC). Integr Cancer Ther. 2020;19:1534735420969818.

10. Howden EJ, Bigaran A, Beaudry R, Fraser S, Selig S, Foulkes S, mfl. Exercise as a diagnostic and therapeutic tool for the prevention of cardiovascular dysfunction in breast cancer patients. European Journal of Preventive Cardiology. 2019;26(3):305–15.

11. Cornette T, Vincent F, Mandigout S, Antonini MT, Leobon S, Labrunie A, mfl. Effects of home-based exercise training on VO2 in breast cancer patients under adjuvant or neoadjuvant chemotherapy (SAPA): a randomized controlled trial. Eur J Phys Rehabil Med. 2016;52(2):223–32.

12. Ndjavera W, Orange ST, O’Doherty AF, Leicht AS, Rochester M, Mills R, mfl. Exercise-induced attenuation of treatment side-effects in patients with newly diagnosed prostate cancer beginning androgen-deprivation therapy: a randomised controlled trial. BJU Int. 2020;125(1):28–37.

13. Al-Majid S, Wilson LD, Rakovski C, Coburn JW. Effects of Exercise on Biobehavioral Outcomes of Fatigue During Cancer Treatment: Results of a Feasibility Study. Biological Research For Nursing. 2015;17(1):40–8.

14. Allen SK, Brown V, White D, King D, Hunt J, Wainwright J, mfl. Multimodal Prehabilitation During Neoadjuvant Therapy Prior to Esophagogastric Cancer Resection: Effect on Cardiopulmonary Exercise Test Performance, Muscle Mass and Quality of Life-A Pilot Randomized Clinical Trial. Ann Surg Oncol. 2022;29(3):1839–50.

15. Chung WP, Yang HL, Hsu YT, Hung CH, Liu PY, Liu YW, mfl. Real-time exercise reduces impaired cardiac function in breast cancer patients undergoing chemotherapy: A randomized controlled trial. Annals of Physical and Rehabilitation Medicine. 2022;65(2):101485.

16. Hornsby WE, Douglas PS, West MJ, Kenjale AA, Lane AR, Schwitzer ER, mfl. Safety and efficacy of aerobic training in operable breast cancer patients receiving neoadjuvant chemotherapy: A phase II randomized trial. Acta Oncologica. 2014;53(1):65–74.

17. Foulkes SJ, Howden EJ, Haykowsky MJ, Antill Y, Salim A, Nightingale SS, mfl. Exercise for the Prevention of Anthracycline-Induced Functional Disability and Cardiac Dysfunction: The BREXIT Study. Circulation. 2023;147(7):532–45.

18. Schneider C, Ryffel C, Stütz L, Rabaglio M, Suter TM, Campbell KL, mfl. Supervised exercise training in patients with cancer during anthracycline-based chemotherapy to mitigate cardiotoxicity: a randomized-controlled-trial. Front Cardiovasc Med. 4. desember 2023;10:1283153.

19. Scott JM, Lee J, Herndon JE, Michalski MG, Lee CP, O’Brien KA, mfl. Timing of exercise therapy when initiating adjuvant chemotherapy for breast cancer: a randomized trial. Eur Heart J. 2023;44(46):4878–89.

20. Kirkham AA, Pituskin E, Mackey JR, Grenier JG, Ian Paterson D, Haykowsky MJ, mfl. Longitudinal Changes in Skeletal Muscle Metabolism, Oxygen Uptake, and Myosteatosis During Cardiotoxic Treatment for Early-Stage Breast Cancer. Oncologist. 2022;27(9):e748–54.

21. West MA, Baker WC, Rahman S, Munro A, Jack S, Grocott MP, mfl. Cardiopulmonary exercise testing has greater prognostic value than sarcopenia in oesophago-gastric cancer patients undergoing neoadjuvant therapy and surgical resection. J Surg Oncol. 2021;124(8):1306–16.

22. Thomson IG, Wallen MP, Hall A, Ferris R, Gotley DC, Barbour AP, mfl. Neoadjuvant therapy reduces cardiopulmunary function in patients undegoing oesophagectomy. Int J Surg. 2018;53:86–92.

23. Jack S, West MA, Raw D, Marwood S, Ambler G, Cope TM, mfl. The effect of neoadjuvant chemotherapy on physical fitness and survival in patients undergoing oesophagogastric cancer surgery. Eur J Surg Oncol. 2014;40(10):1313–20.

24. Navidi M, Phillips AW, Griffin SM, Duffield KE, Greystoke A, Sumpter K, mfl. Cardiopulmonary fitness before and after neoadjuvant chemotherapy in patients with oesophagogastric cancer. Br J Surg. 2018;105(7):900–6.

25. Groehs RV, Negrao MV, Hajjar LA, Jordão CP, Carvalho BP, Toschi‐Dias E, mfl. Adjuvant Treatment with 5‐Fluorouracil and Oxaliplatin Does Not Influence Cardiac Function, Neurovascular Control, and Physical Capacity in Patients with Colon Cancer. Oncologist. 2020;25(12):e13475.

26. Culos-Reed S, Leach HJ, Capozzi LC, Easaw J, Eves N, Millet GY. Exercise preferences and associations between fitness parameters, physical activity, and quality of life in high-grade glioma patients. Support Care Cancer. 2017;25(4):1237–46.

27. Wiestad TH, Raastad T, Nordin K, Igelström H, Henriksson A, Demmelmaier I, mfl. The Phys-Can observational study: adjuvant chemotherapy is associated with a reduction whereas physical activity level before start of treatment is associated with maintenance of maximal oxygen uptake in patients with cancer. BMC Sports Sci Med Rehabil. 2020;12:53.

28. Howden EJ, Foulkes S, Dillon HT, Bigaran A, Wright L, Janssens K, mfl. Traditional markers of cardiac toxicity fail to detect marked reductions in cardiorespiratory fitness among cancer patients undergoing anti-cancer treatment. Eur Heart J Cardiovasc Imaging. 2021;22(4):451–8.

29. Dillon HT, Foulkes S, Horne-Okano YA, Kliman D, Dunstan DW, Daly RM, mfl. Rapid cardiovascular aging following allogeneic hematopoietic cell transplantation for hematological malignancy. Front Cardiovasc Med. 2022;9:926064.

30. Baracos VE, Urtasun RC, Humen DP, Haennel RG. Physical Fitness of Patients with Small Cell Lung Cancer. Clinical Journal of Sport Medicine. 1994;4(4):223–7.

31. Koelwyn GJ, Lewis NC, Ellard SL, Jones LW, Gelinas JC, Rolf JD, mfl. Ventricular-Arterial Coupling in Breast Cancer Patients After Treatment With Anthracycline-Containing Adjuvant Chemotherapy. The Oncologist. 2016;21(2):141–9.

32. Beaudry RI, Kirkham AA, Thompson RB, Grenier JG, Mackey JR, Haykowsky MJ. Exercise Intolerance in Anthracycline‐Treated Breast Cancer Survivors: The Role of Skeletal Muscle Bioenergetics, Oxygenation, and Composition. The Oncologist. 2020;25(5):e852–60.

33. Kirkham AA, Campbell KL, McKenzie DC. Comparison of aerobic exercise intensity prescription methods in breast cancer. Med Sci Sports Exerc. 2013;45(8):1443–50.

34. Jones LW, Haykowsky M, Peddle CJ, Joy AA, Pituskin EN, Tkachuk LM, mfl. Cardiovascular risk profile of patients with HER2/neu-positive breast cancer treated with anthracycline-taxane-containing adjuvant chemotherapy and/or trastuzumab. Cancer Epidemiol Biomarkers Prev. 2007;16(5):1026–31.

35. Crowgey T, Peters KB, Hornsby WE, Lane A, McSherry F, Herndon JE, mfl. Relationship between exercise behavior, cardiorespiratory fitness, and cognitive function in early breast cancer patients treated with doxorubicin-containing chemotherapy: a pilot study. Appl Physiol Nutr Metab. 2014;39(6):724–9.

36. Khouri MG, Hornsby WE, Risum N, Velazquez EJ, Thomas S, Lane A, mfl. Utility of 3-dimensional echocardiography, global longitudinal strain, and exercise stress echocardiography to detect cardiac dysfunction in breast cancer patients treated with doxorubicin-containing adjuvant therapy. Breast Cancer Research and Treatment. 2014;143(3):531–9.

37. Beaudry RI, Haykowsky MJ, MacNamara JP, Tucker WJ, Rao R, Haley B, mfl. Cardiac mechanisms for low aerobic power in anthracycline treated, older, long-term breast cancer survivors. Cardiooncology. 2022;4(4):8.

38. Kirkham AA, Haykowsky MJ, Beaudry RI, Grenier JG, Mackey JR, Pituskin E, mfl. Cardiac and skeletal muscle predictors of impaired cardiorespiratory fitness post-anthracycline chemotherapy for breast cancer. Scientific Reports. 2021;11(1):14005.

39. Jones LW, Haykowsky M, Pituskin EN, Jendzjowsky NG, Tomczak CR, Haennel RG, mfl. Cardiovascular Reserve and Risk Profile of Postmenopausal Women After Chemoendocrine Therapy for Hormone Receptor–Positive Operable Breast Cancer. The Oncologist. 2007;12(10):1156–64.

40. Yu AF, Flynn JR, Moskowitz CS, Scott JM, Oeffinger KC, Dang CT, mfl. Long-term Cardiopulmonary Consequences of Treatment-Induced Cardiotoxicity in Survivors of ERBB2-Positive Breast Cancer. JAMA Cardiol. 2020;5(3):309–17.

41. Long TM, Lee F, Lam K, Wallman KE, Walwyn TS, Choong CS, mfl. Cardiovascular Testing Detects Underlying Dysfunction in Childhood Leukemia Survivors. Med Sci Sports Exerc. 2020;52(3):525–34.

42. Cramer L, Hildebrandt B, Kung T, Wichmann K, Springer J, Doehner W, mfl. Cardiovascular function and predictors of exercise capacity in patients with colorectal cancer. J Am Coll Cardiol. 2014;64(13):1310–9.

43. Reding KW, Brubaker P, D’Agostino R, Kitzman DW, Nicklas B, Langford D, mfl. Increased skeletal intermuscular fat is associated with reduced exercise capacity in cancer survivors: a cross-sectional study. Cardiooncology. 2019;5:3.

44. Tonorezos ES, Snell PG, Moskowitz CS, Eshelman-Kent DA, Liu JE, Chou JF, mfl. Reduced Cardiorespiratory Fitness in Adult Survivors of Childhood Acute Lymphoblastic Leukemia. Pediatr Blood Cancer. 2013;60(8):1358–64.

45. Ness KK, Plana JC, Joshi VM, Luepker RV, Durand JB, Green DM, mfl. Exercise Intolerance, Mortality, and Organ System Impairment in Adult Survivors of Childhood Cancer. JCO. 2020;38(1):29–42.

46. Caru M, Samoilenko M, Drouin S, Lemay V, Kern L, Romo L, mfl. Childhood Acute Lymphoblastic Leukemia Survivors Have a Substantially Lower Cardiorespiratory Fitness Level Than Healthy Canadians Despite a Clinically Equivalent Level of Physical Activity. Journal of Adolescent and Young Adult Oncology. 2019;8(6):674–83.
